# Dendritic Cell Vaccine Harboring Inactivated Mycobacteria Induces Immune Protection Against Tuberculosis in Murine Models and is Well Tolerated in Humans

**DOI:** 10.1002/smsc.202400355

**Published:** 2024-12-18

**Authors:** Zhidong Hu, Xuhui Liu, Jing Wang, Ling Gu, Zhenyan Chen, Lu Xia, Heng Yang, Jinchuan Xu, Xuejiao Huang, Huiling Wang, Shuihua Lu, Xiao‐Yong Fan

**Affiliations:** ^1^ Shanghai Public Health Clinical Center Shanghai Institute of Infectious Diseases and Biosecurity Fudan University Shanghai 201508 China; ^2^ National Clinical Research Center for Infectious Disease The Third People's Hospital of Shenzhen Southern University of Science and Technology Shenzhen 518112 Guangdong China

**Keywords:** dendritic cells, macrophages, T cells, tuberculosis, vaccines

## Abstract

The limited success of tuberculosis (TB) control measures reflects the inadequacy of Bacille Calmette‐Guérin (BCG), the only licensed TB vaccine. There is a recent resurgence of interest in intravenous administration of BCG. However, direct injection of live BCG bacteria into the bloodstream of human beings is not likely to be practical due to safety concerns. In this study, it is showed that debris of BCG‐infected macrophages induces activation and maturation of dendritic cells (DCs) in vitro, and an intravenous DCs vaccine phagocytosing noninfective cell debris induces robust antigen‐specific T‐cell immune responses and immune protection against *Mycobacterium tuberculosis* infection in murine models. Further, an investigator‐initiated clinical trial shows the safety of a DCs vaccine harboring the noninfective *Mycobacterium vaccae* vaccine. Infusions of naive DCs and DCs harboring *Mycobacterium vaccae* are well tolerated and safe in six active TB patients. Tests of the peripheral blood mononuclear cells of a patient who receives two doses of DCs vaccine infusion show enhanced secretion of IFN‐γ, IL‐2, IL‐17, and TNF‐α in both CD4 and CD8 T cells. The study provides evidence that DC‐based vaccines harboring inactivated mycobacteria can expand T‐cell immune responses in TB‐infected mice and are well tolerated in patients with active TB disease.

## Graphical abstract

A dendritic cell vaccine harboring cell debris of Bacille Calmette‐Guérin‐infected macrophages induces T‐cell immunity and immune protection against *Mycobacterium tuberculosis* infection in murine models; similarly, a dendritic cell vaccine harboring *Mycobacterium vaccae* is well tolerated and expands T‐cell immune responses in active tuberculosis patients.

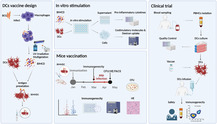

## 1 Introduction

Tuberculosis (TB) is caused by respiratory infection with *Mycobacterium tuberculosis* (*Mtb*) and, every year, is the leading cause of death from a single infectious agent, only being surpassed by COVID‐2019 during the 2019–2021 pandemic. An estimated 10.6 million people fell ill with TB in 2022, with 1.3 million deaths.^[^ ^1^ ^]^ Bacille Calmette‐Guérin (BCG) is the only available vaccine against TB, with more than 4 billion BCG vaccinations administered to date.^[^ ^2^ ^]^ BCG saves lives, especially when given in early life, by inducing both pathogen‐specific immunity^[^ ^3^ ^]^ and nonspecific trained innate immunity,^[^ ^4^, ^5^ ^]^ but it has not stopped the TB epidemic. Protection against the pulmonary disease in adults that is responsible for transmission is generally inadequate.^[^ ^6^ ^]^ The immune mechanisms are not yet fully understood and are under intense investigation. Novel vaccines or immunization strategies are urgently needed and many potential options are under active evaluation.

Most novel strategies use a subunit‐based approach, taking selected antigenic components and finding the best way to deliver them. BCG is a live virulence‐attenuated pathogenic bacterial vaccine, and a large part of the rationale for using selected components is the avoidance of the anti‐immunity and tissue‐destructive effects of residual bacterial products and functions that were needed for successful pathogenesis and were retained during virulence attenuation. However, by modifying BCG or by changing the mode of delivery, the induction of protective immunity can be enhanced.

In 1928, the intradermal (i.d.) route of administration of BCG was found to be more reliable compared with the oral route^[^ ^7^ ^]^ and continues to be used today. However, this route fails to generate sufficient long‐lasting immunological memory in the lungs, which has been proposed as one of the possible reasons for inadequate protection.^[^ ^8^, ^9^ ^]^ The i.d. route could result in suboptimal migration of bacteria to the draining lymph nodes and minimal encounters with antigen‐presenting cells (APCs) in the lungs.^[^ ^10^, ^11^ ^]^ The concept of alternative vaccination routes for BCG was revived as early as the 1970s.^[^ ^12^, ^13^ ^]^ A recent resurgence of interest in the intravenous (i.v.) administration of BCG has followed demonstration of excellent protection in nonhuman primates; the i.v. route of BCG immunization protected 9 out of 10 rhesus macaques against the *Mtb* challenge, in contrast to the limited protection afforded by i.d., mucosal, and other routes of delivery.^[^ ^14^ ^]^ The enhanced early innate immune responses and adaptive CD4 T‐cell immunity in the airway were found to correlate with improved protection following i.v. BCG.^[^ ^15^, ^16^ ^]^ However, direct injection of live BCG bacteria into the bloodstream of human beings is not likely to be practical due to safety concerns, especially in immunocompromised individuals and people infected with nontuberculous mycobacteria and latent TB.^[^ ^17^, ^18^ ^]^

As an alternative approach, the mycobacterium itself can be modified to enhance its protective efficacy. BCG has retained a capacity to render T‐cell priming suboptimal by impairing dendritic cells (DCs) maturation and antigen presentation.^[^ ^19^, ^20^, ^21^ ^]^ It does this by inhibiting the apoptosis process of macrophages, interrupting the antigen presentation by DCs, and weakening the induction of antigen‐specific adaptive immune responses through various mechanisms.^[^ ^22^, ^23^, ^24^, ^25^, ^26^ ^]^ A recombinant BCG vaccine, VPM1002 (BCG Δ*ureC:hly*), that was genetically engineered to increase autophagy, apoptosis‐mediated antigen presentation, and cross‐presentation pathways in APCs gave more robust protective immune responses^[^ ^27^, ^28^, ^29^, ^30^, ^31^ ^]^ and the vaccine is now in a phase III clinical efficacy trial against TB (NCT04351685).

A third approach to improving the vaccine efficacy could be the use of APCs that have been preloaded with BCG ex vivo. DCs that phagocytosed apoptotic macrophages loaded with mycobacterial antigen have shown enhanced T‐cell immune function,^[^ ^32^ ^]^ and autophagy‐inducing DCs that were infected with BCG have given enhanced T‐cell immunity‐mediated protection against *Mtb* infection.^[^ ^33^ ^]^ These observations suggest that targeting DCs also might overcome the bottleneck of suboptimal T‐cell immune protection induction by the TB vaccine.

Herein, we show that the UV‐sterilized BCG‐infected macrophage cell debris (BIMCD) induced activation and maturation of DCs in vitro. This prompted us to assess DCs harboring the BIMCD (BCG‐infected macrophage‐harboring DCs [BIMHDC]) as a vaccine. We tested immunogenicity and efficacy in inducing immune protection against *Mtb* infection in murine models and then performed an investigator‐initiated clinical trial to assess the safety of i.v. infusions of a similar DCs vaccine harboring inactivated *Mycobacterium vaccae*,^[^ ^34^, ^35^ ^]^ an immunomodulator licensed in China. The DCs vaccine harboring inactivated *M. vaccae* showed a well‐tolerated profile in six patients with active TB. In patient 6, who received two doses of the DCs vaccine, the intracellular cytokine staining (ICS) assay showed that the secretion of IFN‐γ, IL‐2, IL‐17, and TNF‐α in both CD4 and CD8 T cells was enhanced, and this was associated with an increased expression of T‐cell activation markers. Taken together, the observations in our study provide evidence that DC‐based vaccines harboring inactivated mycobacteria antigens can expand T‐cell immune responses, not only in *Mtb*‐infected murine models but also in patients with active TB infection.

## 2 Results

### 2.1 The BIMCD‐Induced Activation and Maturation of DCs In Vitro

**Figure 1:**
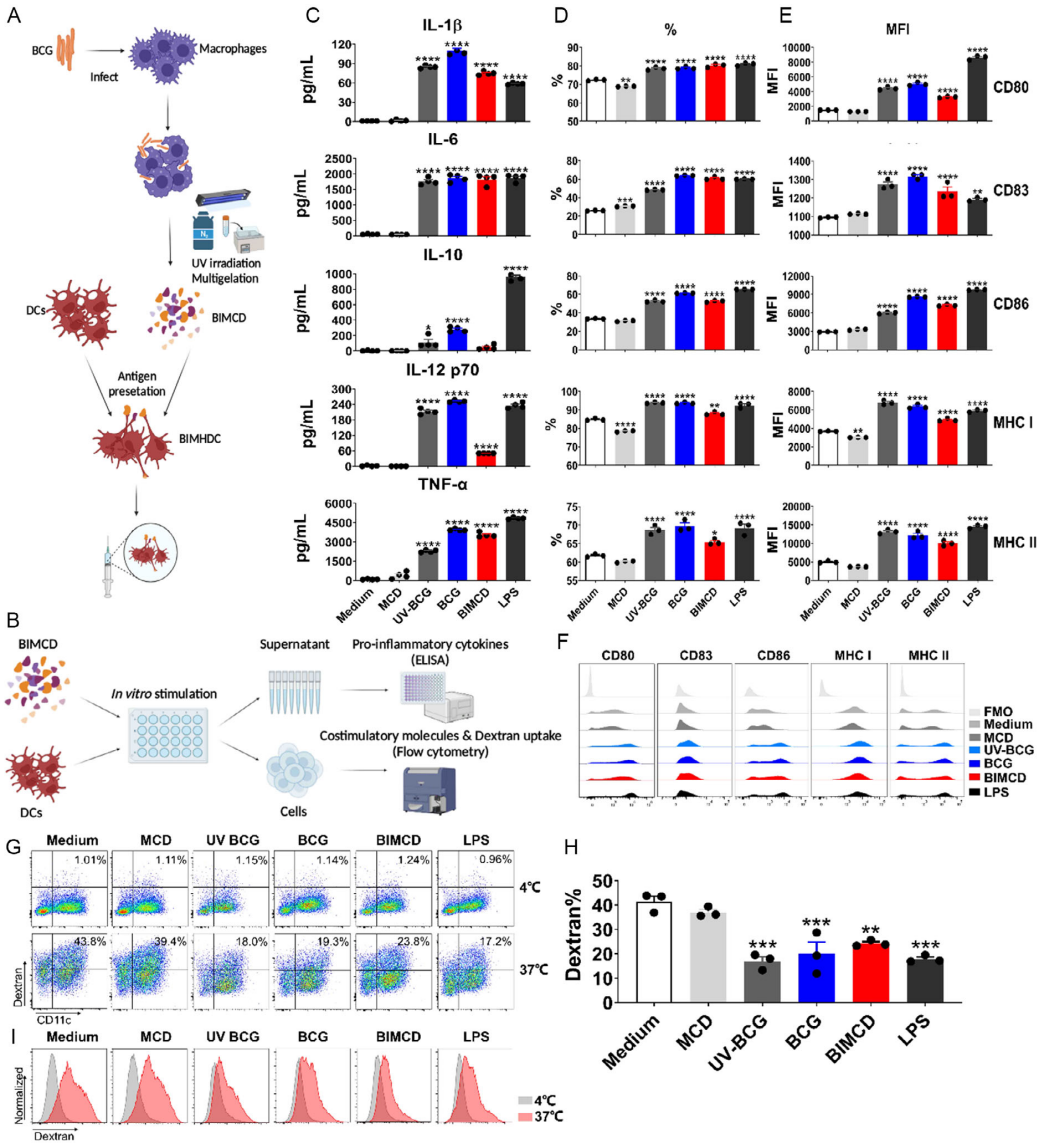
BIMCD‐induced activation and maturation of mouse DCs. A) The flow diagram of the BIMHDC vaccine preparation. BCG‐infected macrophages were disrupted, sterilized by UV irradiation and cell debris (BIMCD) was incubated with ex vivo differentiated immature BMDCs for 24 h to provide DCs harboring BIMCD vaccine (BIMHDC). B) The flow diagram assaying the in vitro stimulation of BMDCs by BIMCD. UV‐inactivated BCG, live BCG, blank macrophage cell debris (MCD), medium alone, and LPS were used as comparators. C–F) BIMCD‐enhanced production of proinflammatory cytokines and expression of costimulatory and MHC molecules on BMDCs. (C) The secretion of IL‐1β, IL‐6, IL‐10, IL‐12p70, and TNF‐α in the culture medium was measured by ELISA (*n* = 4, one‐way ANOVA). (D–F) The expression of surface markers CD80, CD83, CD86, MHC class I, and MHC class II was determined by flow cytometry (*n* = 4, one‐way ANOVA). The frequency (D) and median fluorescence intensity (E) of the positive cells are shown. A representative flow cytometric histogram is shown in (F). G–I) The endocytic activity of activated DCs was determined by dextran uptake. The stimulated DCs were incubated with dextran‐FITC at 37 °C for 30 min and the uptake was assessed by flow cytometric analysis (*n* = 3, one‐way ANOVA). Incubation at 4 °C was used as the negative control. Representative flow cytometric plots of dextran‐FITC and CD11c‐PE cells are shown in (G), and the proportion of dextran^+^ on CD11c^+^ cells are shown as histograms (H). Representative flow cytometric histogram of dextran^+^ on CD11c^+^ cells, which are compared between 4 °C (negative control) and 37 °C, are shown in (I). These results are representative of three independent experiments with three or four replicate wells per group. Values are expressed as mean ± SEM. *p < 0.05, **p < 0.01, ***p < 0.001, and ****p < 0.0001.

Considering direct i.v. inoculation of live BCG bacteria into the bloodstream of human beings is not practical due to safety reasons, we initially asked whether it is feasible to induce robust antigen‐specific immune protection against *Mtb* through noninfective BCG. However, it was reported that inactivated BCG failed to afford enough protection.^[^ ^36^, ^37^, ^38^ ^]^ Given the central role that DCs play in the induction of antigen‐specific adaptive T‐cell response, we utilized bone marrow‐derived DCs (BMDCs) as a DCs vaccine to enhance the immunogenicity of noninfectious BCG. Considering phagocytosis of whole apoptotic cells carrying mycobacteria antigens promotes a potentially protective immune response,^[^ ^32^ ^]^ we prepared cell debris from BCG‐infected macrophages (BIMCD) by multigelation of BCG‐infected bone marrow‐derived macrophages (BMDMs) and used UV irradiation to inactive the residual bacteria. As shown in **Figure** **1**A, by using a combination of BIMCD and naïve BMDCs, we obtained a vaccine composed of DCs containing inactivated BCG‐infected macrophages (BIMHDC vaccine).

To be immunogenic, DCs must mature to express a critical amount of antigen presentation makers and costimulatory molecules.^[^ ^39^ ^]^ To evaluate the immunoregulatory effect of BCG‐infected BMDM cell debris, we first stimulated BMDCs by using BIMCD and measured the expression of phenotypic markers of DCs maturation (Figure 1B). The BMDCs were treated with BIMCD stimulation, with lipopolysaccharide (LPS) as a positive control and phosphate buffered saline (PBS) as a negative control, respectively. Both UV‐inactivated BCG and live BCG induced the secretion of proinflammatory cytokines (IL‐1β, IL‐6, IL‐10, IL‐12, and TNF‐α) from DCs, although UV‐inactivated BCG was less effective than live BCG (Figure 1C). Notably, the blank MCD failed to promote the production of proinflammatory cytokines, but BIMCD significantly enhanced the secretion of these cytokines, and the effect was noninferior to that of intact UV‐inactivated BCG, although lower than live BCG in inducing IL‐1β, IL‐10, and IL‐12 (Figure 1C). Flow cytometric analysis also revealed that BIMCD significantly augmented the expression of CD80, CD83, CD86, MHC class I molecules, and MHC class II molecules, evidenced by the percentage of positive cells (Figure 1D,F), median cell fluorescence intensity (MFI; Figure 1E,F), and integrated MFI (Figure S1A, Supporting Information). The gating strategy of flow cytometric analysis is shown in Figure S1B, Supporting Information.

Considering that the capacity of DCs to take up an antigen is known to decrease after DCs maturation, we next investigated endocytic activity by exposing the DCs to fluorescein isothiocyanate dextran. Double‐positive cells (CD11c^+^dextran‐FITC^+^) were decreased in number among DCs treated with BIMCD, UV‐BCG, live BCG, and LPS at 37 °C, compared with medium and blank MCD controls. This confirmed an endocytosis reduction associated with enhancement of functional maturity by BIMCD (Figure 1G–I). Taken together, these results indicated that BIMCD phenotypically and functionally activated the DCs in vitro.

### 2.2 The BIMHDC‐Induced Antigen‐Specific Th1 Cellular Immune Responses in Mice

**Figure 2:**
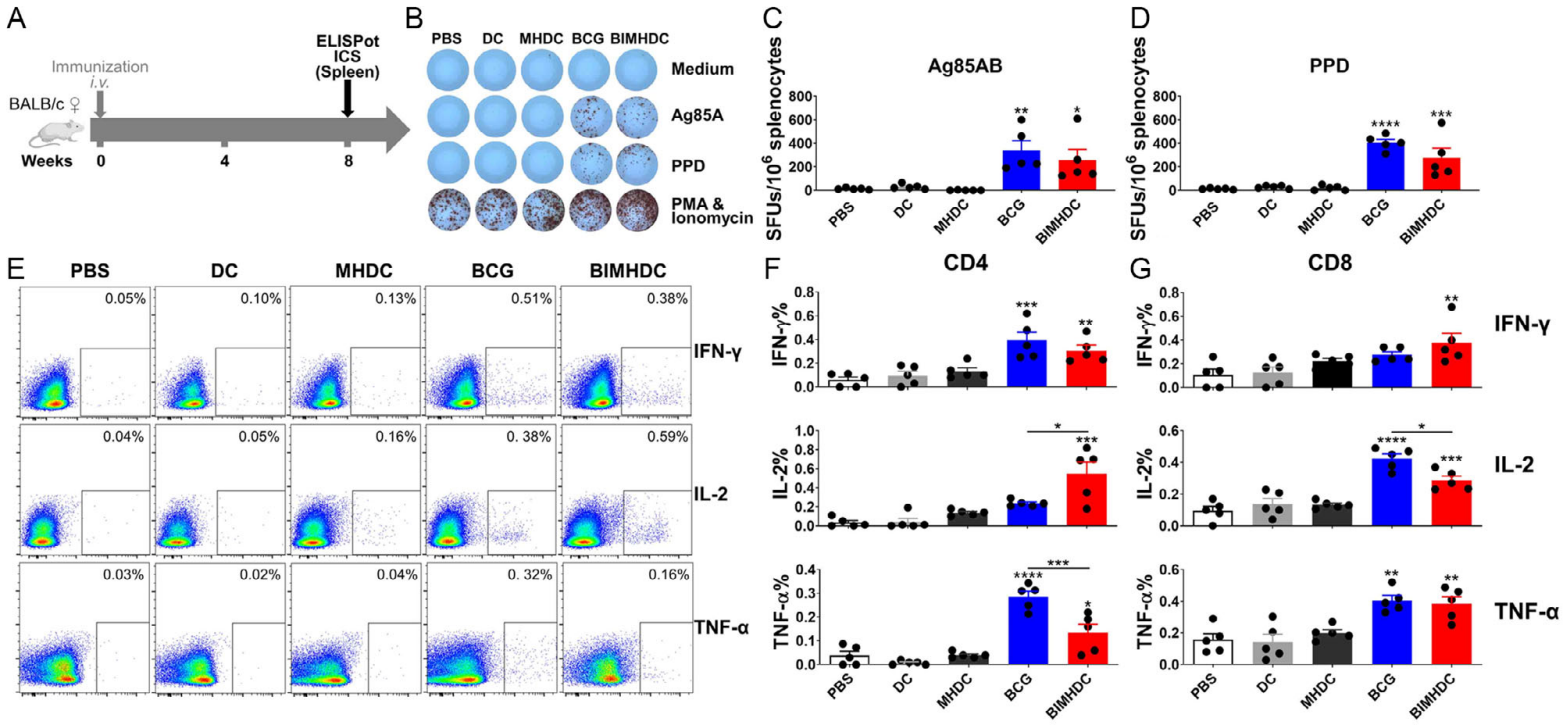
BIMHDC vaccine‐induced antigen‐specific Th1 cellular immune responses in the spleens of vaccinated mice. A) Vaccination and detection schedule. Cellular immune responses to Ag85AB peptides and PPD of *Mtb* were assayed eight weeks after vaccination. B) Representative splenocyte IFN‐γ responses revealed by ELISPOT assay (*n* = 5, one‐way ANOVA). Quantified responses to Ag85AB and PPD peptides are shown in C,D), respectively. E–G) Flow cytometric analysis of cytokine production. Eight weeks after vaccination, splenocytes were harvested and stimulated with the peptides in the presence of monensin and brefeldin A, and then analyzed for intracellular cytokine production by ICS assay (*n* = 5, one‐way ANOVA). Representative flow cytometric plots of the IFN‐γ, IL‐2, and TNF‐α staining in T cells are shown in (E), and the proportion of cells producing these cytokines among CD4 (F) and CD8 (G) T cells in response to PPD are shown as histograms. The results are representative of two independent experiments with five mice per group. Values are expressed as mean ± SEM. *p < 0.05, **p < 0.01, ***p < 0.001, and ****p < 0.0001.

We nex*t* tested whether the BIMCD‐matured DCs could stimulate antigen‐specific adaptive immune responses. Mice were vaccinated i.v. with the DC vaccine (BIMHDC, containing 1 × 10^6^ DCs and 1 × 10^6^ BCG‐infected MCD with multiplicity of infection [MOI] = 1), with PBS, blank DCs, blank macrophage‐harboring DCs (MHDC, containing 1 × 10^6^ DCs and 1 × 10^6^ blank MCD) as negative controls, and live BCG (1 × 10^6^ colony‐forming units [CFU]) as a positive control (**Figure** **2**A). Eight weeks after vaccination, the spleens and lungs were harvested and digested to provide single‐cell suspensions. These cells were stimulated with either Ag85AB peptides which are dominant antigens of *Mtb*, or purified protein derivative (PPD) of *Mtb*. As expected, the IFN‐γ enzyme‐linked immunospot assay (ELISPOT) assay showed that blank DCs and MHDC failed to induce *Mtb*‐specific adaptive immune responses. In contrast, the BIMHDC induced robust Ag85AB‐ and PPD‐specific IFN‐γ responses in the splenocytes, which was comparable to or modestly (not significantly) lower than the live BCG vaccine (Figure 2B–D).

Th1 cells (those producing IFN‐γ, IL‐2, and TNF‐α after PPD stimulation) were determined by flow cytometry (the gating strategy is shown in Figure S2, Supporting Information). In spleen cells, the frequencies of CD4 T cells and CD8 T cells expressing either IFN‐γ, IL‐2, or TNF‐α after PPD stimulation were significantly higher in mice vaccinated with BIMHDC, compared to control mice vaccinated with PBS, blank DCs, and MHDC (Figure 2E–G). IFN‐γ/IL‐2 secretion in the CD4 T cells (Figure 2F) and IFN‐γ/IL‐2/TNF‐α secretion in the CD8 T cells (Figure 2G) were comparable between live BCG and BIMHDC groups. However, the TNF‐α responses in the CD4 T cells were significantly lower in the BIMHDC group compared to the live BCG group (Figure 2F).

**Figure 3:**
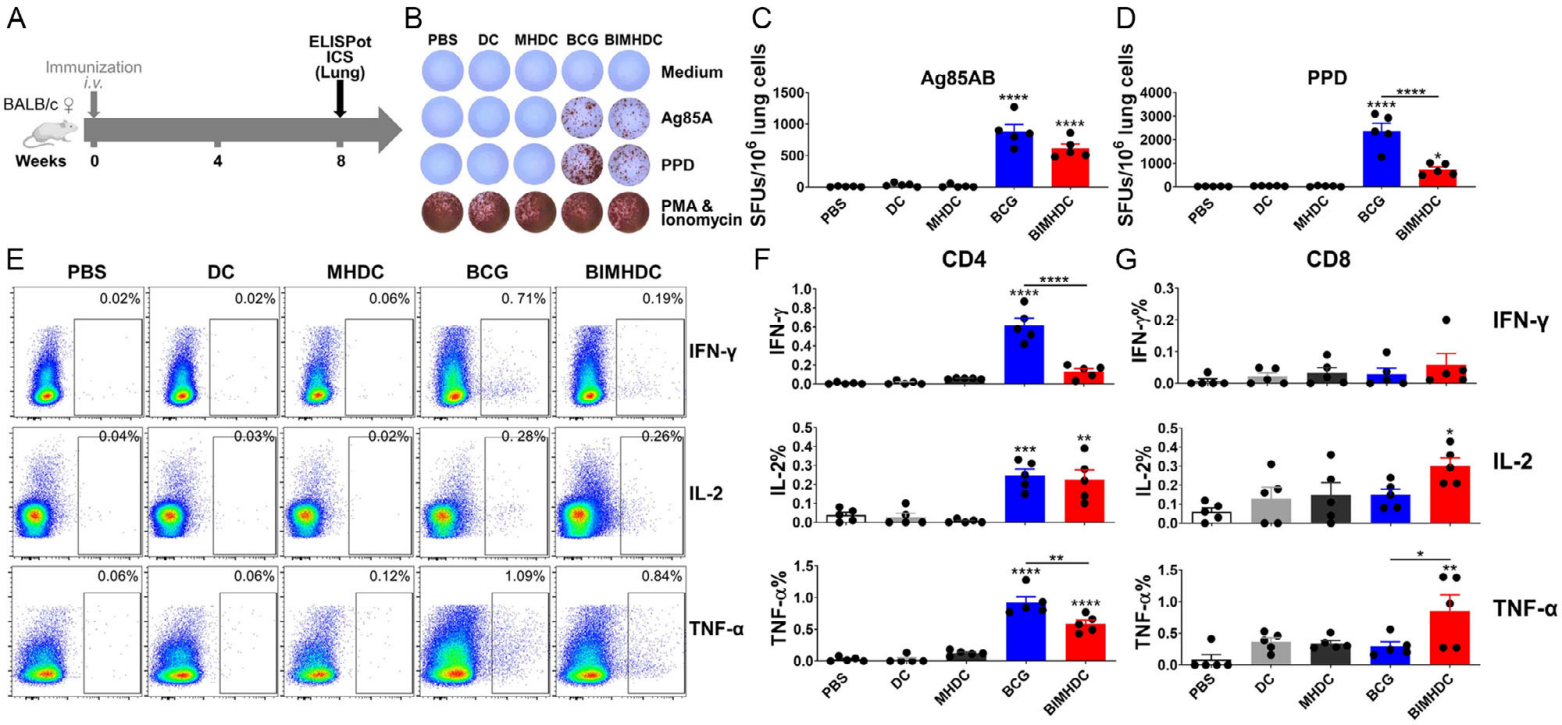
BIMHDC vaccine‐induced antigen‐specific Th1 cellular immune responses in the lungs of vaccinated mice. A) Vaccination and detection schedule. Cellular immune responses to Ag85AB peptides and PPD of *Mtb* were assayed eight weeks after vaccination. B) Representative IFN‐γ responses revealed by ELISPOT assay (*n* = 5, one‐way ANOVA). Quantified responses to Ag85AB and PPD peptides are shown in C,D), respectively. (E–G) Flow cytometric analysis of cytokine production by lung cells of immunized mice. Eight weeks after vaccination, lung cells were harvested and stimulated with the peptides in the presence of monensin and brefeldin A, and then analyzed for intracellular cytokine production by ICS assay (*n* = 5, one‐way ANOVA). Representative flow cytometric plots of IFN‐γ, IL‐2, and TNF‐α staining in T cells are shown in (E), and the proportion of cells producing these cytokines among CD4 (F) and CD8 (G) T cells in response to PPD are shown as histograms. The results are representative of two independent experiments with five mice per group. Values are expressed as mean ± SEM. *p < 0.05, **p < 0.01, ***p < 0.001, and ****p < 0.0001.

In lung cells (**Figure** **3**A), the BIMHDC had induced robust Ag85AB‐ and PPD‐specific IFN‐γ responses in ELISPOT assays (Figure 3B–D), although the PPD‐specific IFN‐γ responses in the BIMHDC group were significantly lower than in the live BCG vaccine group (Figure 3D). Consistent with the ELISPOT assays, the ICS assay showed that the frequencies of CD4 T and CD8 T cells expressing IL‐2 and TNF‐α after PPD stimulation were significantly higher in mice vaccinated with BIMHDC compared to mice vaccinated with PBS, blank DCs, and MHDC (Figure 3E–G), although in contrast both the BCG and BIMHDC vaccines failed to induce detectable IFN‐γ responses in CD8 T cells (Figure 3G). Notably, compared to the live BCG group, BIMHDC induced modestly (not significantly) lower levels of TNF‐α responses and significantly lower levels of IFN‐γ responses in the CD4 T cells (Figure 3F), as well as modestly but not significantly higher levels of IL‐2/TNF‐α responses in the CD8 T cells (Figure 3G). In addition, both the BCG and the BIMHDC vaccines failed to elicit robust antigen‐specific IL‐17 responses (Figure S3, Supporting Information).

Taken together, these results suggest that BIMHDC induced high levels of *Mtb*‐specific multifunctional Th1 cellular immune responses.

### 2.3 The BIMHDC Induced Immune Protection Against *Mtb* Infection

**Figure 4:**
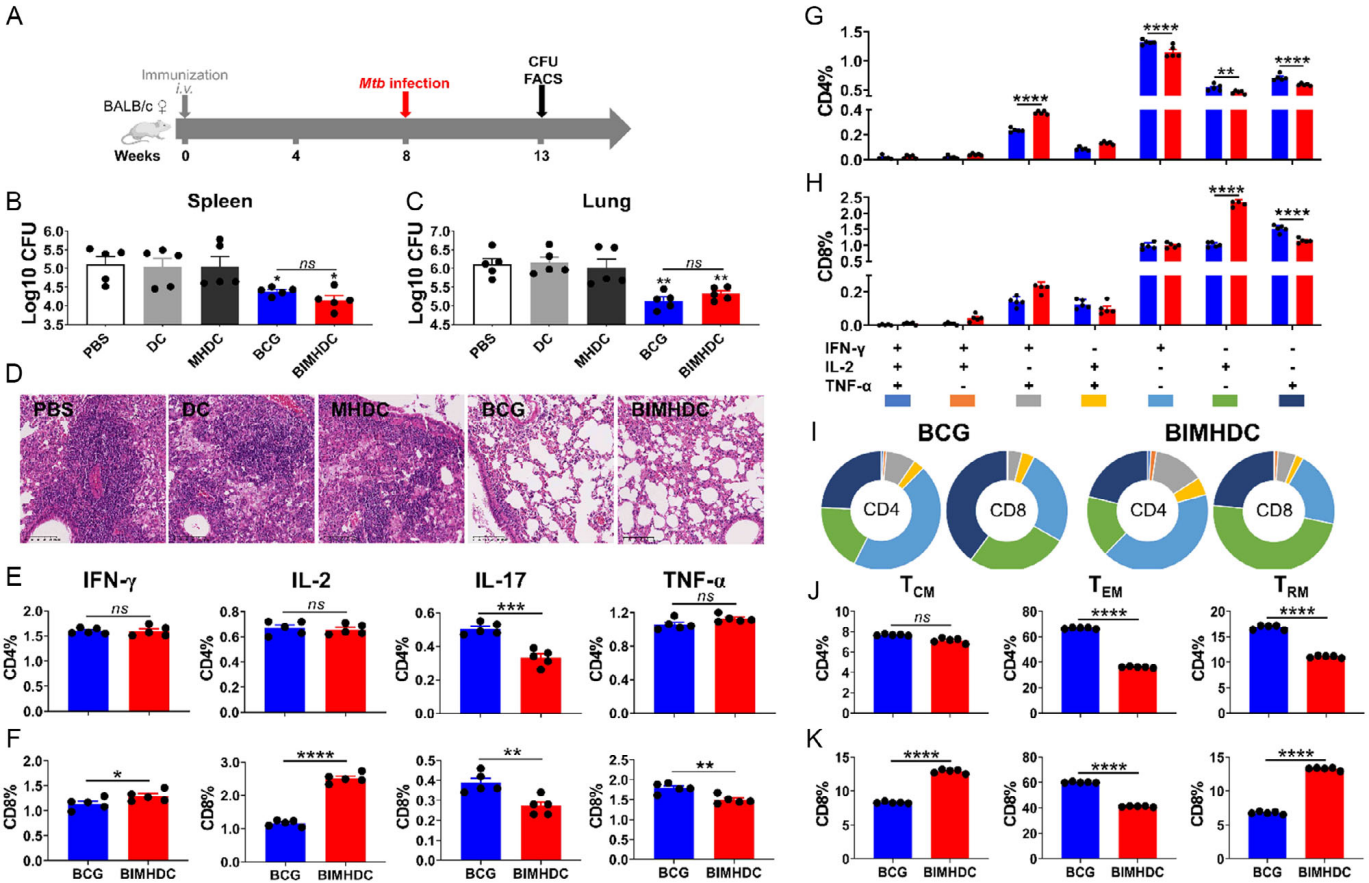
Vaccine protective efficacy and *Mtb*‐specific recall T‐cell immune responses against *Mtb* challenge in mice. A) Vaccination, infection, and detection schedule. Mice received blank DCs, DCs loaded with blank macrophage debris (MHDC), BCG, or DCs loaded with debris from BCG‐infected macrophages (BIMHDC) by i.v. injection, and then were challenged with virulent *Mtb* H37Rv strain 8 weeks later. Five weeks postinfection, the numbers of live bacteria in homogenates of the spleens B) and lungs C) were counted as CFU after 3 weeks of incubation on 7H11 agar and transformed as log_10_ (*n* = 5, one‐way ANOVA). D) Tissue sections from the right superior lung lobes were stained for histopathology with H&E. Representative histological appearances are shown in (D), show ×200, scale bar = 100 μm. E–K) The recall T‐cell immune responses in the lung cells 5 weeks postinfection. The largest lobe of lung tissue from each mouse in a group was pooled and digested to liberate single lung cells by using collagenase IV and DNase I. The cells were then stimulated with PPD in the presence of monensin and brefeldin A, then analyzed for surface marker expression and intracellular cytokine production by ICS assay. The proportions of CD4 (E) and CD8 (F) T cells producing IFN‐γ, IL‐2, and TNF‐α are shown as histograms (*n* = 5, nonpaired *t*‐test). The frequencies of the seven cell subpopulations, based on the possible combinations of expression of IFN‐γ, IL‐2, and TNF‐α, are shown for CD4 (G) and CD8 (H) T cells (*n* = 5, two‐way ANOVA), and pie chart analysis is shown in (I). The memory phenotypes in CD4 (J) and CD8 (K) T cells are shown (*n* = 5, nonpaired *t*‐test). T_CM_ and T_EM_ were defined as CD44^+^CD62L^+^ and CD44^+^CD62L^−^, respectively. CD4 T_RM_ was defined as CXCR3^+^KLRG1^−^, and CD8 T_RM_ was defined as CD69^+^CD103^+^. The results are representative of two independent experiments with five mice per group. Values are expressed as mean ± SEM. *p < 0.05, **p < 0.01, ***p < 0.001, ****p < 0.0001, and ns, no significant difference.

To directly evaluate the BIMHDC‐induced protective efficacy against *Mtb* infection in a murine model, we immunized mice as indicated above and determined the bacterial loads over five weeks post‐*Mtb* challenge (**Figure** **4**A). The bacillary loads in the lungs and spleens of BIMHDC‐vaccinated mice were significantly reduced compared to the negative control groups of PBS, blank DCs, and MHDC (Figure 4B,C). A similar reduction in bacterial counts was also observed in the BCG group (Figure 4B,C). In addition, decreased inflammation was observed in hematoxylin and eosin (H&E)‐stained lung sections derived from BCG‐ and BIMHDC‐vaccinated mice (Figure 4D). Thus, the inactivated BIMHDC induced protection against *Mtb* infection.

### 2.4 The Recall Immune Responses Post‐*Mtb* Infection

Considering that the BCG vaccine is a live bacterium and BIMHDC is a mixture of inactivated cell debris, it is reasonable to assume that the immune responses they induce may be mediated by different mechanisms. Therefore, we further characterized the antigen‐specific immune responses underlying the enhanced protection observed in BIMHDC‐ and BCG‐vaccinated mice. First, the levels of intracellular cytokines in PPD‐stimulated lung cells were quantified at 5 weeks postchallenge. The BIMHDC‐vaccinated mice showed decreased accumulation of activated IL‐17 CD4 and CD8 T cells and TNF‐α‐secreting CD8 cells compared with BCG‐vaccinated mice (Figure 4E,F). In contrast, the IFN‐γ and IL‐2 responses in CD8 T cells were significantly higher in BIMHDC‐vaccinated mice (Figure 4F), suggesting that the DCs carrying BCG‐infected MCD had superior ability to cross‐present antigens to CD8 T cells.

A poly‐functionality analysis of the stimulated T cells showed that, compared with BCG, the BIMHDC enhanced the proportion of dual‐positive IFN‐γ^+^TNF‐α^+^ cells in both CD4 and CD8 T‐cell populations, as well as the monopositive IL‐2^+^ CD8 T cells (Figure 4G,I). In contrast, BCG typically caused enhancements in the proportions of monopositive IFN‐γ, IL‐2, and TNF‐α cells among CD4 T cells and of TNF‐α monopositive cells among CD8 T cells compared with BIMHDC (Figure 4G,I).

Finally, we compared the memory phenotypes of T cells in the lung between BIMHDC‐ and BCG‐vaccinated mice. The gating strategy of flow cytometric analysis is shown in Figure S4, Supporting Information. BCG vaccination led to significantly higher levels of CD4 and CD8 T cells with CD44^+^CD62^−^ effector memory phenotype (T_EM_), which is consistent with other reports showing that BCG‐induced T‐cell responses are mediated by T_EM_ responses (Figure 4J,K). In contrast, the BIMHDC vaccination induced significantly higher levels of CD44^+^CD62^+^ central memory phenotype (T_CM_) in CD8 T cells (Figure 4K). Interestingly, using CXCR3^+^KLRG1^−^^[^ ^40^, ^41^ ^]^ and CD69^+^CD103^+^^[^ ^41^, ^42^ ^]^ as markers of lung tissue‐resident memory (T_RM_) in CD4 and CD8 T cells, respectively, we found that BIMHDC induced significantly higher levels of T_RM_ responses in the lung compared with BCG immunization (Figure 4J,K). This result further suggested that the BCG‐infected cell debris favored cross‐presentation of the antigens to CD8 T cells.

Taken together, the above data support the hypothesis that BIMHDC can generate potent DC‐based TB vaccines in murine models.

### 2.5 Preliminary In Vitro Stimulation Experiments Showed DCs Addition Enhanced the *Mtb* Antigen‐Induced T‐cell Immune Response of TB Patient's PBMCs

**Figure 5:**
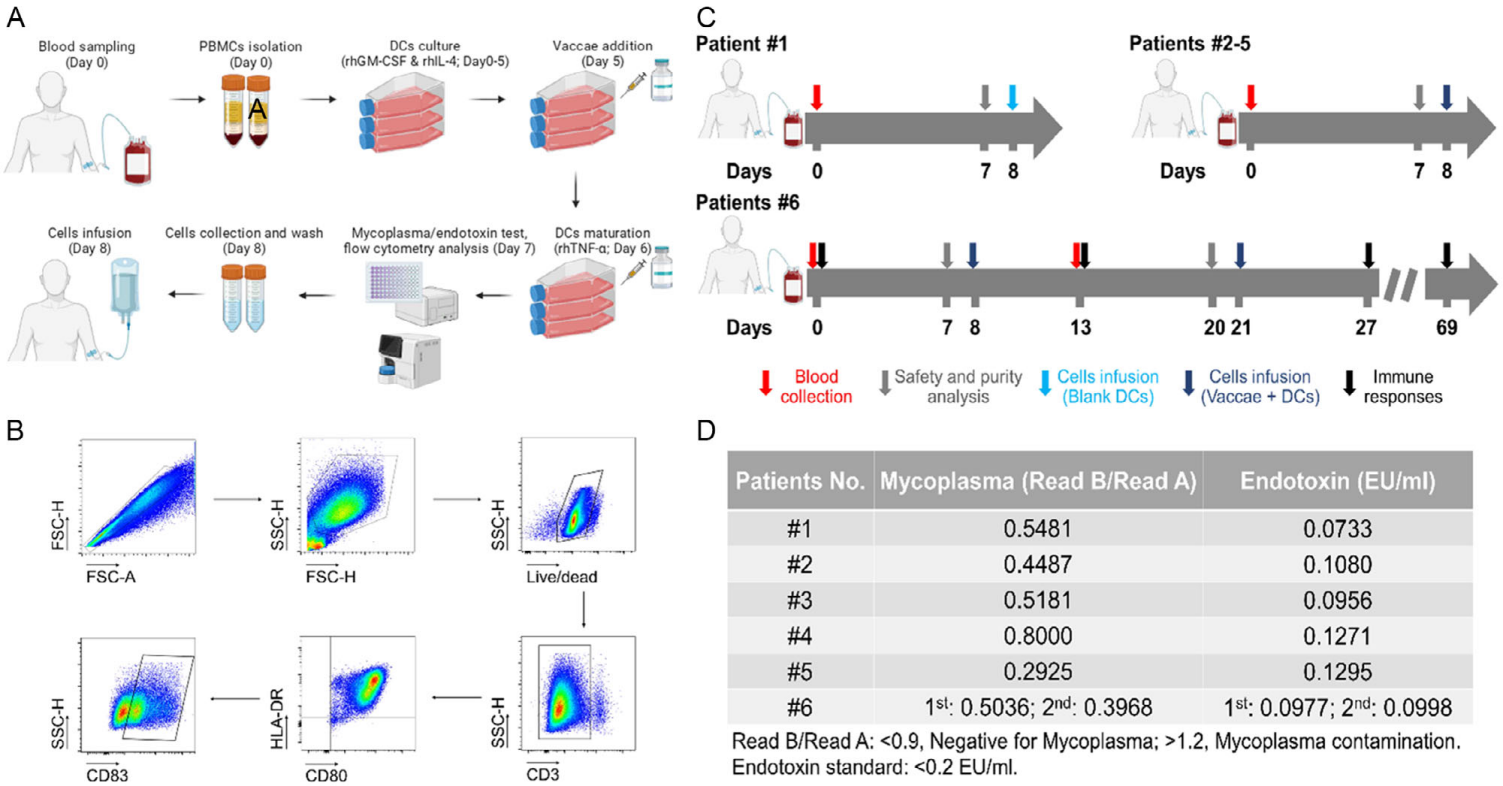
Assessment of the clinical safety of DCs harboring inactivated *M. vaccae* vaccine. A) Brief flow diagram of human DCs differentiation. The PBMCs were isolated from freshly heparinized blood and cultured in DCs medium in the presence of rhGM‐CSF and rhIL‐4. Maturation was induced by rhTNF‐α after antigen loading. Differentiation of the DCs was confirmed by flow cytometric analysis of live cell percentage, DCs purity, and DCs maturation. B) Representative of the flow cytometric analysis of DCs products that was performed before every infusion. C) Flow diagram of the treatment of each patient. Patient 1 received blank DCs. Patients 2–5 received a single dose of DCs infusion. Patient 6 received two doses of DCs infusion. The number of DCs per infusion dose are shown in Table 1. D) Mycoplasma and endotoxin detection assays were performed before every infusion, as a part of the quality controls of DCs preparation.

The results from animal studies promoted us to perform a clinical study using DCs vaccine harboring noninfective mycobacteria antigens in human being. To start, we first performed a preliminary experiment to determine whether human peripheral blood mononuclear cell (PBMC)‐derived DCs addition could enhance *Mtb* antigen‐induced immune responses in TB patients. DCs were differentiated from the patient's PBMCs in the presence of rhGM‐CSF and rhIL‐4, and maturation was induced with rhTNF‐α one day after incubation with antigen (**Figure** **5**A). The differentiated DCs were confirmed by flow cytometric analysis (Figure 5B) and light microscopy (Figure S5, Supporting Information). Through in vitro stimulation experiment, DCs harboring *Mtb* antigens showed a stronger ability to induce antigen‐specific immune responses compared with direct antigen stimulation of PBMCs from four individual active TB patients (Figure S6, Supporting Information), consistent with our contention that DCs could be used to enhance antigen‐specific cellular immune responses against TB in human beings.

### 2.6 The Safety of a DC Vaccine Harboring Inactivated *M. Vaccae* in a Phase I Clinical Trial

Considering the complexity of BIMCD and in compliance with the Good Clinical Practice guidelines and local regulatory requirements, we selected the inactivated *M. vaccae* vaccine Vaccae, a licensed immunomodulator in China, for investigating potential safety concerns. The induction of mycobacteria‐specific immune responses by the DCs vaccine was assumed to be benefit. A scheme of the study design is shown in Figure 5C. A total of six patients were enrolled in the phase I clinical trial (**Table** **1**). Patient 1 received one infusion of 1 × 10^7^ blank DCs 8 days after phlebotomy, patients 2–5 received one infusion of DCs harboring inactivated *M. vaccae* vaccine 8 days after blood drawing, and patient 6 received two doses of infusion of DCs harboring inactivated *M. vaccae* vaccine at an interval of 13 days. The exact numbers of DCs per infusion dose are shown in Table 1. The quality controls, including mycoplasma detection (Figure 5D), endotoxin detection (Figure 5D), live cells percentage (Figure 5B), DCs purity (Figure 5B), and maturity rate (Figure 5B), were performed before every infusion. The DCs products with live/dead cell percentage higher than 90%, DCs purity more than 60%, maturity rate higher than 50%, and negativity for mycoplasma/endotoxin were considered qualified for infusion. For patient 6, at day 0, day 13, day 27, and day 69, the additional blood was collected to profile the immune responses (Figure 5C).

**Table 1:** The clinical characteristics of the subjects participating in the clinical trial.

Patient nos.a)	Gender	Age	Diagnosis	Chemotherapy	Cell therapy	Cell dosage (*10^7^)	Safety		Treatment outcome			Follow‐up	
							AE	Relevance	Clinical symptoms	Bacteriological conversion	Imaging	Last follow‐up	Stop medication
#1	Male	27	MDR‐TB with combined with drug‐induced liver damage, and NTM infections	ZEMfxCsCmPto	Naïve DCs	2	Manic episodeb)	Irrelevant	Disappearance	Yes	Significant improvement	May, 2020	No
#2	Male	45	Vertebral XDR‐TB, delayed wound healing postoperation, initial treatment failure	LzdZPtoAm	DCs + *Mycobacterium vaccae*	5.3	No	–	Disappearance	Yes	Significant improvement	Feb, 2021	Yes
#3	Male	44	TB with persistent hemoptysis, drug‐induced liver damage	HEMfx	DCs + *Mycobacterium vaccae*	2	No	–	Disappearance	Yes	Improvement	Feb, 2021	Yes
#4	Male	33	Vertebral TB, delayed wound healing postoperation, drug‐induced liver damage	HRE	DCs + *Mycobacterium vaccae*	1	No	–	Disappearance	Yes	Significant improvement	May, 2020	No
#5	Male	21	Pulmonary, spinal TB combined with Aspergillus infection, delayed wound healing postoperation	HEZMfxLzd + antifungal drug (voriconazole)	DCs + *Mycobacterium vaccae*	1	No	–	Disappearance	Yes	Improvement	Feb, 2021	Yes
#6	Female	22	Primary immunodeficiency combined with pulmonary, cervical and hilar lymphatic TB, repeated treatment without healing	HRftEZAmLfx	DCs + *Mycobacterium vaccae*	1 (first dose) + 1.6 (second dose)	No	–	Disappearance	Yes	Improvement	Nov, 2020	Yes

a) MDR, multidrug resistance; XDR, extensively drug resistance; TB, tuberculosis; DCs, dendritic cells; AE, adverse event; NTM, nontuberculous mycobacteria.

b) The manic episode in patient #1 was associated with a stressful life event. The patient self‐administered antibiotics for TB, was mistaken for a drug user by a neighbor, and had a manic episode during police questioning. With the help of a psychotherapist, the patient's mental condition returned to normal. This manic episode was characterized as a transient stress reaction, possibly related to cycloserine and not related to DCs therapy.

As a result, the mycoplasma and endotoxin tests confirmed the safety profiles of the DCs products (Figure 5D). Blank DCs and DCs harboring inactivated *M. vaccae* vaccine were safely administered to patients 1 and 2–6, respectively. Patient 1 experienced an adverse event (manic episode) that was considered unrelated to DCs treatment (see Table 1 for detail). Patients 2–5 did not experience any adverse events.

### 2.7 The T‐cell Immune Profiles of the Patients Who Received Two Doses of DC Infusion

**Figure 6:**
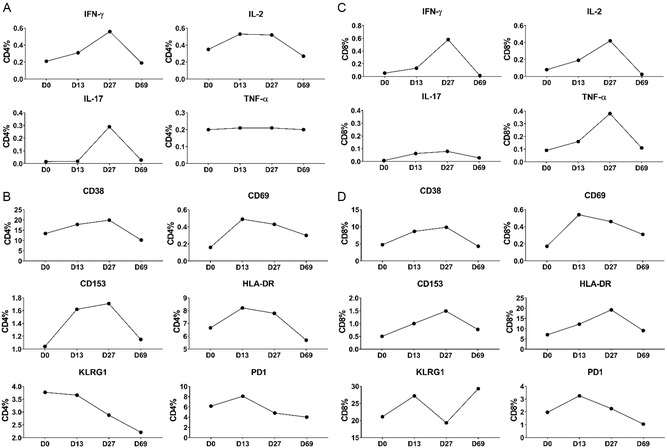
T‐cell immune profiles of patient 6 who received two doses of DCs infusion. The PBMCs were collected on day 0, day 13, day 27, and day 69 and T‐cell immune responses profiled by ICS assay. The intracellular cytokines IFN‐γ, IL‐2, IL‐17, and TNF‐α in CD4 and CD8 T cells are shown in A,C), respectively. The expression profiles of surface markers CD38, CD69, CD153, HLA‐DR, KLRG1, and PD1 on CD4 and CD8 T cells are shown in B,D), respectively.

Considering that T‐cell responses are regarded as the most crucial factor in immune protection against *Mtb* infection, we profiled the T‐cell immune responses by flow cytometry in patient 6, who received two cell infusions (the gating strategy of flow cytometric analysis of intracellular cytokines is shown in Figure S7, Supporting Information). **Figure** **6**A shows that intracellular cytokine CD4 IFN‐γ responses were increased 5 days after the first dose (day 13) compared with baseline levels (day 0), reached a maximum 5 days after the second dose (day 27), and then returned to baseline 48 days after the second dose (day 69). A similar trend was observed in CD4 IL‐2 and IL‐17 responses, although IL‐2 responses reached a maximum after the first dose and kept high after the second dose, and IL‐17 began to increase after the second dose (Figure 6A). In contrast, the TNF‐α responses were not significantly influenced by the cell infusions. In line with the intracellular assays of T‐cell responses, the T‐cell activation markers CD38, CD69, CD153, and HLA‐DR were elevated after cell infusion and returned to baseline levels on day 69 (Figure 6B). The T‐cell exhaustion marker KLRG1 expressed on CD4 T cells kept decreasing after each cell infusion, and the levels of PD1's expression on CD4 T cells also decreased after a slight initial increase after the first cell infusion (Figure 6B). The gating strategy of flow cytometric analysis of surface markers is shown in Figure S8, Supporting Information.

A similar trend was observed in CD8 T cells, in which the content of secretory all cytokines (IFN‐γ/IL‐2/IL‐17/TNF‐α) was enhanced after the first cell infusion and peaked after the second infusion (Figure 6C). The expression levels of T‐cell activation markers on CD8 T cells were also well consistent with the secretion of cytokines. All the surface markers were increased after the first cell infusion, kept on increasing after the second cell infusion, and returned to baseline on day 69, except that the expression of CD69 peaked after the first cell infusion (Figure 6D). Regarding the two T‐cell exhaustion markers, the levels of PD1 expression on CD8 and CD4 T cells were similar, but the expression of KLRG1 was elevated on day 69 (Figure 6D).

Taken together, the promising immune response profiles and safety indicate the feasibility and potential therapeutic impact of using DCs harboring inactivated *M. vaccae* or other mycobacterial antigens as a vaccine or immunotherapy for active TB infection.

## 3 Discussion

The failure of TB control indicates that BCG, the only licensed TB vaccine, is insufficient. Strategies to improve TB vaccination mainly use two approaches: optimizing the current BCG vaccine or developing novel vaccines such as vectored, subunit, and live attenuated vaccines. In the past decades, several novel vector‐based and protein‐adjuvant subunit vaccines were designed as a booster of the BCG vaccine^[^ ^9^, ^43^ ^]^ since most adults who acquire TB worldwide today were BCG‐vaccinated as neonates. However, one of the major bottlenecks in vectored and subunit vaccine design is that *Mtb* expresses ≈4000 proteins.^[^ ^44^ ^]^ It is still controversial which antigens were the most effective ones in inducing immune protection against *Mtb* infection, casting a shadow on vectored and subunit TB vaccines design. Alternatively, the live attenuated *Mtb*‐based vaccine could express broader antigenic panel compared with vectored and subunit TB vaccines, and might be more effective in inducing immune responses closely resembling a natural infection of *Mtb*. However, safety is the primary concern with the potential for attenuation reversal.^[^ ^45^, ^46^ ^]^ Although inactivated *Mtb* vaccines are safer with the ability to present a wide repertoire of antigens to the recipient, they have conventionally conferred limited protection against subsequent infection with virulent mycobacteria, partially due to the important protective antigens are only expressed when the bacteria are metabolically active in the intracellular infection phase.^[^ ^47^ ^]^ Thus, optimizing the current BCG vaccine is still one of the most important pipelines of TB vaccine design.

Although the concept of alternative vaccination routes of BCG was suggested as early as the 1970s,^[^ ^12^, ^13^ ^]^ a resurgence of interest in the BCG i.v. delivery has attracted much attention during the past 3 years in the TB vaccine research field. Notably, in 2020, it was found that i.v. delivery of BCG protected 9 out of 10 macaques from TB disease, with 6 of them even exhibiting sterilizing immunity (undetectable CFU).^[^ ^14^ ^]^ This encouraging result was confirmed by another group, which showed i.v. BCG also protected 9 out of 12 simian immunodeficiency virus‐infected animals without any culturable bacteria detected from tissues.^[^ ^48^ ^]^ These studies provide a model to identify correlates of protection in *Mtb* control,^[^ ^15^, ^16^, ^49^, ^50^ ^]^ which might lay the groundwork for future studies to develop effective TB vaccine regimens. However, we must realize that direct inoculation of live BCG bacteria into the bloodstream of human beings is not likely to be practical due to safety reasons.

Besides the altered route of vaccination, another approach being actively pursued to improve BCG efficacy is to reverse the mycobacteria‐mediated inhibition of T‐cell priming, which was mediated by impairing DCs maturation and efficient antigen presentation.^[^ ^19^, ^20^, ^21^ ^]^ It was well elucidated that mycobacteria could inhibit the apoptosis process of macrophages, interrupt the antigen presentation of DCs, and then weaken the induction of antigen‐specific adaptive immune responses through various mechanisms.^[^ ^22^, ^23^, ^24^, ^25^, ^26^ ^]^ The recombinant BCG vaccine VPM1002 (BCG *ΔureC:hly*) has been engineered to express the *Listeria monocytogenes* protein listeriolysin O to replace urease C, which inhibits acidification of the phagosome by converting urea to ammonia and preventing phagosome maturation.^[^ ^30^, ^31^ ^]^ VPM1002 enhanced protection by increasing autophagy and apoptosis‐mediated antigen presentation and cross‐presentation pathways,^[^ ^27^, ^28^, ^29^ ^]^ and the phase III clinical efficacy trial against TB is ongoing (NCT04351685).

These studies suggested targeting DCs could enhance TB vaccine‐induced immune protection against infection. Actually, it was well known that DCs are one of the principal defense components that play multifactorial roles in translating innate immunity to adaptive immune responses during *Mtb* infection. However, *Mtb* infection induces a bacteria‐favoring microenvironment by regulating DCs differentiation and function.^[^ ^51^ ^]^ For example, it was reported that *Mtb* delayed T‐cell response by inhibiting the ability of DCs to act as APCs through *Mtb*‐infected neutrophil‐apoptosis inhibition.^[^ ^52^ ^]^ Thus, the accumulation of activated T cells into the lung is delayed, occurring between 14 and 21 days post‐*Mtb* infection.^[^ ^53^, ^54^ ^]^ Similar DC‐targeting immune escape mechanisms for the BCG vaccination also existed.^[^ ^10^, ^55^ ^]^ Thus, enhancing the function of DCs during BCG vaccination will strengthen their antigen‐representing ability and lead to a more robust T‐cell immune response. Griffiths K et al. showed that pulmonary delivery of activated *Mtb* antigen‐primed DCs into recipient mice at the time of exposure can overcome the delay in accumulation of vaccine‐induced CD4 T‐cell responses.^[^ ^56^ ^]^ However, we cannot foresee when *Mtb* exposure happens in the real world. Thus, prestimulating DCs is only therapeutically sound. In this study, we choose DCs adoptive transfer as a carrier of noninfective BCG antigens in murine models. Our observations that infusion of DCs that are preloaded with inactivated mycobacteria antigen is highly immunogenic in vitro (Figure 1), in mice (Figure 2, 3, 4), and in human (Figure 5 and 6) are consistent with the findings from numerous studies that DC‐based vaccines/immunotherapies could raise immunity against cancer and autoimmune diseases.^[^ ^57^, ^58^ ^]^

Particularly, in this study, the BCG vaccine was inactivated after infecting macrophages, which are the target cells and the primary resident niche during mycobacteria early infection. BIMCD stimulated robust  inflammatory responses of DCs, we assume the mechanism is that variously BCG‐derived antigens that were released by the lysis of BCG‐infected macrophages have the stimulation capacity. Importantly, the released antigens in BIMCD are anticipated to be different from directly lysis of live BCG bacteria since the lysis of BCG‐infected macrophages might reserve the important protective antigens that are only expressed in the intracellular infection phase. In addition, during BCG subcutaneous vaccination, some bacteria might be killed, proteasomal degraded, and digested within macrophages; in that case, this BCG‐infected cell debris might not have the chance to be released by macrophage and then be presented by APCs to lymphocytes to induce adaptive immune responses. Herein, we used ex vivo differentiated DCs to phagocytose these potential immunogenic antigens and adoptively transferred them to receipt mice. Thus, these antigens would be directly presented to naïve T cells or B cells to induce immune responses effectively. In mice, we explored the hypothesis that the suboptimal performance of BCG in protecting against TB might be improved by using debris from disrupted BCG‐infected macrophages to provide antigen to DCs for infusion as a vaccine. The response of the DCs to the debris (BIMCD) differed from the response to either live or dead BCG that it stimulated less production of IL‐12 and IL‐10 and slightly less enhancement of surface markers of in vitro activation (Figure 1). Such differences in effect of stimulation with BIMCD and BCG may have contributed to the different outcomes of i.v. immunization with infusion of debris‐stimulated DCs (BIMHDC) and BCG. In addition, although the BIMHDC vaccine showed similar immune protection compared with the live BCG vaccine, the immune response profiles were different between the two groups (Figure 4), further suggesting that the mechanisms of immune protection induced by BIMHDC are different from live BCG vaccines.

Our results are consistent with a study that showed apoptotic BMDMs, which were induced by incubating the macrophage with cell wall extracts of mycobacteria expressing a *Mtb* cell wall glycolipoprotein LpqH, could activate BMDCs by upregulating the inflammatory cytokines and costimulatory molecules.^[^ ^32^ ^]^ The activated DCs further stimulated T‐cell‐mediated immunity and proliferation during in vitro coculture.^[^ ^32^ ^]^ Thus, this study supports the phagocytosis of whole apoptotic cells carrying mycobacterial antigens, which promotes an antigen‐specific immune response. However, no animal study was reported to determine the immunogenicity and protective efficacy of this LpqH‐inducing apoptotic macrophages as a TB vaccine up to now. One possibility is that the immune responses against the *Mtb* lipoprotein LpqH are not strong enough to protect or even detrimental to the host. In this study, we used the BCG‐infected macrophages by assuming that some important protective antigens might only be expressed during the infection process. Thus, DCs phagocytosing these antigen fragments might effectively induce immune responses that could inhibit the acute infection of *Mtb*. A recently published report supports this hypothesis by showing that coadministration of BCG and MK‐2206, an anti‐cancer Akt inhibitor, enhanced BCG‐induced apoptosis of macrophages and resulted in an improvement of BCG‐induced protection in animal models.^[^ ^33^ ^]^ However, considering BCG is a routinely recommended neonatal vaccine, inducing BCG‐induced apoptosis of macrophages in primary vaccination in neonates might cause additional safety problems. In this study, we chose the inactivated BCG‐infected macrophages as antigens, different from BCG vaccine, the response induced by BIMHDC vaccine was biased toward a stronger response in CD8 cells that produced IFN‐γ, IL‐2, and TNF‐α after antigen stimulation (Figure 2 and 3). These are the cells that have been implicated as major effectors of cytotoxicity and killing of *Mtb*. As a result, the protection that was conferred by vaccination with BIMHDC against aerosol challenge infection with *Mtb* was similar to, and not superior to, that from i.v. BCG vaccination (Figure 4). Furthermore, the cellular immunity expressed in the infected lungs was biased toward IL‐2‐driven expansion of CD8 cells with central memory and resident memory phenotypes in BIMHDC‐vaccinated mice and not in BCG‐vaccinated mice (Figure 4). This implies a superior ability to generate long‐lasting protection; this awaits evaluation in future experiments. Importantly, with similar efficacy, the BIMHDC vaccine might be considered superior to live BCG in a clinical context since it carries no risk of producing BCG‐osis. These studies in mice established that it may not be necessary to use intravenous delivery of a living BCG to obtain the most robust protective response; nonliving antigenic fragments from infected macrophages were equally effective when delivered in DCs. Thus, the safety profiles and immune protection afforded by this novel vaccine might provide a new direction on the live i.v. BCG immunization strategy design for potential human use.

Since the infusion of DCs can only have limited application as a prophylactic TB‐preventive procedure, experiments are underway in mice to assess the potential for application as a therapeutic procedure in human. Adjunctive immunotherapy in cases when chemotherapy is failing may be of practical benefit. Meanwhile, to assess extrapolation of these findings in the mouse model to potential clinical application in human we turned to using a commercial product, Vaccae, that is approved for human use as an immune stimulant against TB in China. We demonstrated the safety and immunogenicity of infusions of DCs loaded with this antigen into a small number of TB patients. Thus, our findings provide several pieces of direct scientific evidence that support further exploration of curative approaches, particularly DCs vaccination in treating TB (i.e., drug‐resistant TB as personalized therapy). Firstly, the blank ex vivo‐differentiated DCs and DCs phagocytosing noninfective *M. vaccae* vaccine showed safety and tolerability after cell infusion into the active TB patients. Second, the cell infusion of DCs phagocytosing noninfective *M. vaccae* vaccine enhanced the T‐cell immune responses, which was reflected by the increase of intracellular cytokines secretion in T cells and enhanced expression of activation markers on T cells. More studies that replace the noninfective *M. vaccae* vaccine with other vaccines are warranted to further improve T‐cell immunity.

The current study has several limitations. First, to what extent the quality of the response is dependent upon a particular profile of antigens produced in infected macrophages remains unknown. Further detailed investigations exploring the exactly different mycobacteria antigens expressed in different infection phases might help answering this question. Second, for safety and feasibility concerns, DCs phagocytosing BCG‐infected MCD was not used for cell infusion in human study. Instead, a licensed immunomodulator Vaccae was used. Further investigations might use other potent antigens to replace the noninfective *M. vaccae* vaccine. Although the i.v. immunization of BIMHDC is anticipated to be much safer than live BCG vaccine, the side effects or complications, such as cytokine storm, allergic reaction, or even thrombogenesis, might be inevitable if large‐scale clinically applicated. Thus, we must be cautious on the clinical usage of BIMHDC, and again, exploring the contribution of more exactly mycobacteria antigens expressed in different infection phases on BIMHDC‐induced protection might lead to a safer and “cleaner” vaccine. Third, in the current investigator‐initiated trial, only six patients were included, only one patient received a two‐round cell infusion, and the immune responses were monitored. A phase II clinical study is warranted to better evaluate the therapeutic efficiency of DCs infusion. Fourth, patient enrollment was limited to active TB patients. Further investigations could be expanded to test the effect of DCs infusion in other *Mtb*‐infective disease statuses, such as latent TB infection or recurrent TB, to test whether DCs vaccine could eliminate the dormant or persistent bacteria.

In summary, our study showed that DCs vaccine phagocytosing BCG‐infected MCD induced antigen‐specific T‐cell immune responses and immune protection against *Mtb* infection in murine models and provided evidence that noninfective *M. vaccae*‐harboring DCs infusion is safe and efficacious in treating active TB patients.

## 4 Experimental Section

4.1

4.1.1

##### Ethics Statement, Clinical Trial Design, Approval, and Process

All animal studies were approved by the Institutional Animal Care and Use Committee and were performed in accordance with the Laboratory Animal Ethical Board of Shanghai Public Health Clinical Center. An investigator‐initiated clinical trial (ChiCTR‐INR‐16 009 606) was also conducted to evaluate the safety and feasibility of DCs therapy in patients with TB. The clinical study was approved by the Ethical Committee of Shanghai Public Health Clinical Center (2016‐S050‐01), and informed consent was obtained from all subjects. Herein, we report part of the results of this clinical trial. All enrolled patients were clinically diagnosed as active TB on the basis of sputum/effusion smear/culture positivity and confirmed by radiological findings, clinical syndromes, and polymerase chain reaction amplification. Exclusion criteria included a history of organ dysfunction; lymphoma or leukemia; diabetes; tumor; pregnancy; coronary heart disease; positive for HIV antibodies, hepatitis B surface antigen, or hepatitis C virus RNA. The age of the enrolled patients ranged between 21 and 45 years old.

##### The Isolation and Differentiation of Murine BMDCs and BMDMs

The BMDCs and BMDMs were generated as previously described with modifications.^[^ ^59^, ^60^ ^]^ Briefly, the bone marrow cells harvested from the femur and tibia of naïve Balb/c mice were diluted at a concentration of 2 × 10^5^ cells mL^−1^. For BMDCs, the bone marrow cells were cultured in RPMI 1640 medium (Hycolne, SH30022.01B) containing 10% fetal bovine serum (FBS, Gibco, 16 000‐044), 1% penicillin & streptomycin, and supplemented with 20 ng mL^−1^ recombinant mouse granulocyte‐macrophage colony‐stimulating factor (rmGM‐CSF; Peprotech, 315‐03) at 37 °C in 5% CO_2_. Four days after the initial culture, the culture medium was semireplenished with fresh RPMI 1640 medium supplemented with rmGM‐CSF and incubated until day 7. On day 7, nonadherent cells were collected, washed, and counted for further use. For BMDMs, the bone marrow cells were cultured in DMEM/F12 medium (Corning, 10‐092‐CVa) containing 10% FBS, 1% penicillin & streptomycin, and supplemented with 100 U/mL recombinant mouse macrophage colony‐stimulating factor (rmM‐CSF; Peprotech, 250‐05) at 37 °C in 5% CO_2_. Four days after the initial culture, cells were supplemented with fresh DMEM/F12 medium containing 100 U mL^−1^ rmM‐CSF and incubated until day 7. At day 7, adherent cells were washed with PBS and digested in Cellstripper nonenzymatic cell dissociation solution (Corning, 25‐056‐CI) for 5 min at 37 °C. The cells were collected, washed, and counted for further use.

##### Bacterial Strains

The BCG Danish strain and *Mtb* H37Rv strain (ATCC 27 294) used in this study were stocked in our laboratory. They were grown at 37 °C in liquid Middlebrook 7H9 broth (BD Difco, 271 310) supplemented with 10% (v/v) oleic acid–albumin–dextrose–catalase enrichment (OADC; BD Difco, 212 352), 0.05% (v/v) Tween‐80, and 0.5% (v/v) glycerol, or cultured on solid 7H11 agar (BD BBL, 212 203) supplemented with 10% OADC and 0.5% glycerol.

##### Preparation of BIMCD

The BMDMs were seeded into flat‐bottom 6‐well cell culture plates at a concentration of 2 × 10^6^ cells/well and infected with BCG at the MOI = 1 for 24 h at 37 °C; the cells were then washed to get rid of nonphagocytosed bacteria. BMDMs without BCG infection were used as the negative control. The cells were washed and resuspended in PBS and subjected to multigelation at least 5 times (quick‐freezing in liquid nitrogen and thawing at room temperature) to lyse the cells and release the cell‐digested BCG fragments and then subjected to UV sterilization for 1 h. Then, the sterilized cell debris was harvested and stocked at −80 °C for further use.

##### The Inflammatory Cytokines Secretion by In Vitro Stimulated DCs

The BMDCs were seeded into flat‐bottom 24‐well cell culture plates at a concentration of 1 × 10^6^ cells/well and incubated the BIMCD or blank (control) MCD with cell counts at 1:1 (counted before multigelation) or infected with UV‐inactivated BCG or live BCG at an MOI = 1 for 24 h at 37 °C, with the medium as a negative control and LPS as a positive control. The culture supernatant was collected to detect IL‐1β (eBioscience, 88‐7013‐86), IL‐6 (eBioscience, 88‐7064‐86), IL‐10 (eBioscience, 88‐7105‐86), IL‐12p70 (eBioscience, 88‐7121‐86), and TNF‐α (eBioscience, 88‐7324‐86) by enzyme‐linked immunosorbent assay (ELISA) kits according to manufacturer's instruction.

##### The Expression of Surface Molecules by In Vitro Stimulated DCs

The BMDCs were stimulated as described above and then were harvested and washed with fluorescence‐activated cell sorting (FACS) staining buffer (PBS containing 2% FBS). A mixture of antibodies against surface markers (CD80, CD83, CD86, MHC I, and MHC II; detailed information on antibodies is provided below) was added to stain the cells for 30 min at 4 °C. The cells were washed and resuspended in FACS staining buffer and then analyzed by flow cytometry LSRFortessa (BD Biosciences) within 2 h.

##### In Vitro Dextran Uptake Ability of In Vitro Stimulated DCs

The BMDCs were seeded at a concentration of 2 × 10^5^ cells/well at a U‐bottom 96‐well cell culture plate. They were stimulated with different stimulants, with the medium as negative control and LPS as positive control. Then, the cells were equilibrated at 37 °C for 45 min, and then were incubated with FITC fluorescein‐conjugated dextran (Sigma, FD40S) at a concentration of 1 mg mL^−1^. Then, 4 °C precooled medium was added to stop the reaction. The cells were washed 3 times, stained with anti‐CD11c antibodies (detailed information on antibodies is provided below), and then analyzed by flow cytometry LSRFortessa (BD Biosciences). The nonspecific binding of dextran to DCs was determined by incubating DCs with FITC‐conjugated dextran at 4 °C and subtracted from the binding values.

##### Mice, Immunization, and Mtb Challenge

Specific pathogen‐free (SPF) female Balb/c mice aged 6–8 weeks were purchased from Shanghai SLAC Laboratory Animal Co., Ltd. and maintained with food and water ad libitum under SPF conditions until sacrifice. For DCs vaccination, the BMDCs were cultured as described above and mixed with the stocked BIMCD with cell counts at 1:1 (counted before multigelation, containing BCG with MOI = 1) at a concentration of 4 × 10^6^ DCs/mL and named BIMHDC. Identical amounts of blank (no BCG) macrophage cell debris‐harboring DCs (MHDC) provided a negative control. The mice were vaccinated with one dose of DCs vaccines (1 × 10^6^ cells/mouse, 250 μL, i.v.) through a lateral tail vein. The PBS and blank DCs were used as negative controls, and the BCG Danish vaccine (1 × 10^6^ CFU/mice, 250 μL, i.v.) was used as the positive control. For the BIMHDC immunization, live BCG bacteria cannot be detected by 7H11 agar plating 1 week after immunization in the lung, spleen, and blood of vaccinated mice (data not shown), confirming live bacteria is not existed in this DCs vaccine. Eight weeks after immunization, the spleens and lungs were aseptically removed to assess antigen‐specific T‐cell immune responses. The vaccinated mice were aerosol‐challenged with virulent *Mtb* H37Rv strain at a dose of ≈100 CFU per lung by an inhalation exposure system (Glas‐Col) in a biosafety level 3 containment animal facility. At 5 weeks postinfection, the spleens and lungs were sampled for the determination of mycobacterial burden by plating homogenates onto Middlebrook 7H11 agar plates supplemented with 10% OADC and antibiotic mixture (40 U mL^−1^ polymyxin B, 4 mg mL^−1^ amphotericin, 50 mg mL^−1^ carbenicillin, and 2 mg mL^−1^ trimethoprim).^[^ ^61^ ^]^

##### Single‐Cell Isolation in Mouse Experiments

Mouse spleens were harvested aseptically and mechanically disrupted. The splenocytes were filtered through mesh gauze, and then red blood cells were lysed with red cell lysis buffer. Lungs were aseptically harvested and then minced with scissors. The tissue pieces were digested for 45 min at 37 °C with 200 μg/mL of DNase I (Roche, 11 284 932 001) and 1 mg mL^−1^ of collagenase IV (Invitrogen, 17 104‐019) in RPMI‐1640 medium containing 10% FBS and 1% penicillin & streptomycin. The digest was filtered through a 70 μm cell strainer (Fisher Scientific) by gently squashing with a syringe plunger and then the red blood cells were lysed. The splenocytes and lung lymphocytes were washed twice, counted, and resuspended in RPMI‐1640 medium containing 10% FBS and 1% penicillin & streptomycin.

##### IFN‐γ ELISPOT

The ELISPOT assays were performed according to the manufacturer's instruction (IFN‐γ ELISPOT kit, BD Biosciences, 551 083), and the spot‐forming cells were counted after adding the AEC Substrate (BD Biosciences, 551 951).

##### Intracellular Cytokine Staining in Mouse Experiments

The freshly harvested single lung cells or splenocytes were stimulated with peptide pools of Ag85A/Ag85B (5 μg mL^−1^) or *Mtb* PPD (10 μg mL^−1^) in the U bottom 96‐well plates at 37 °C and 5% CO_2_ for 1 h, and an additional 10 h in the presence of Brefeldin A (BD Biosciences, 555 029) and Monensin (BD Biosciences, 554 724). Then, the stimulated cells were stained with live/dead fluorochrome for 30 min at 4 °C, followed by washing by PBS, and stained with a mixture of antibodies against surface markers (CD3, CD4, CD44, CD62L, CD69, CD103, CXCR3, and KLRG1; the detailed information of the antibodies is provided below) for 30 min at 4 °C, followed by washing, fixation, and permeabilization with fix/perm buffer (BD Biosciences) for 20 min at 4 °C. The fixed cells were then mixed with antibodies against intracellular cytokines (IFN‐γ, IL‐2, IL‐17, and TNF‐α, as detailed below) and then incubated for another 30 min at 4 °C before analysis by flow cytometry LSRFortessa (BD Biosciences).

##### Histopathological Analysis

The right superior lobes of *Mtb*‐infected lungs were fixed with 4% neutral formaldehyde, embedded in paraffin, serially sectioned in a thickness of 4 μm, stained with H&E, and photographed using an Olympus CKX41 microscope fitted with a DP20 camera connected to a computer.

##### Human DCs Isolation and Culture with Vaccae

The PBMCs were isolated from freshly heparinized blood by Ficoll density centrifugation. The isolated cells were washed with PBS 3 times, counted, and resuspended in RPMI 1640 medium at a concentration of 1 × 10^6^ cells mL^−1^. The PBMCs were seeded in T175 cell culture flasks in a volume of 30 mL and incubated in a humidified 37 °C incubator containing 5% CO_2_ for 1 h. The supernatant was discarded to remove nonadherent cells and then the flasks were washed twice with 30 mL of medium. The adherent cells were cultured in GMP Dendritic Cell Medium (CellGenix, 20 901‐0500) with 50 ng mL^−1^ recombinant human GM‐CSF (rhGM‐CSF; R&D, 215‐GM‐050) and 100 ng mL^−1^ recombinant human IL‐4 (rhIL‐4; R&D, 204‐IL‐010) in a humidified 37 °C incubator containing 5% CO_2_. After 4 day incubation, half of the culture medium was changed into fresh DCs medium containing rhGM‐CSF and rhIL‐4. On day 5, Vaccae was added (22.5 μg). On day 6, 500 U mL^−1^ recombinant human TNF‐α (rhTNF‐α; R&D, 210‐GMP‐02M) was added to stimulate the maturation of DCs. On day 7, the cell culture was harvested and centrifuged at 800 × *g* for 6 min. The supernatant was collected to test whether the DCs products were contaminated with mycoplasma and endotoxin, as described below, and the cells were collected to assess the live cell percentage, purity, and maturation by using flow cytometric analysis, as described below. On day 8, the cell culture was harvested into a 50 mL tube and centrifuged at 800 × *g* for 6 min. The supernatant was discarded, and the cells were washed with physiological saline to remove cell debris: 2 times with 40 mL by centrifugation at 800 × *g* for 6 min and once with 40 mL by centrifugation at 200 × *g* for 10 min. The cells were resuspended in 250 mL of physiological saline solution for infusion to active TB patients through venous transfusion within 2 h.

##### Mycoplasma Detection in Human DCs Products

The mycoplasma contamination was assessed with a MycoAlert mycoplasma detection kit (Lonza, LT07‐218). Briefly, 100 μL cell culture supernatant and 100 μL reagent solution were incubated in a tube for 5 min at room temperature, the luminescence was measured as Read A on a microplate reader, and then the regent was incubated with substrate solution for an additional 10 min at room temperature; the luminescence was measured as Read B. Read B was divided by Read A to produce the ratio. If the ratio was <0.9, it was considered negative for mycoplasma; if the ratio was >1.2, it was considered mycoplasma contaminated; if the ratio was between 0.9 and 1.2, it should be retested. Preparations assayed as mycoplasma negative (<0.9) were deemed qualified for DCs infusion.

##### Endotoxin Detection in Human DCs Products

The endotoxin content was determined by a chromogenic Tachypleus amebocyte lysate assay kit (Xiamen Bioendo Technology, EC64405). Briefly, the pyrogen‐free microplate was prewarmed in a 37 °C incubator for 5 min, and then 50 μL cell culture supernatant or assay standards with different concentrations of endotoxin were added in the plate, with blank buffer as the negative control. The plates were then incubated with 50 μL Tachypleus amebocyte lysate for 13 min at 37 °C. Chromogenic substrate solution (100 μL) was added and incubated for an additional 6 min. The reaction was stopped by adding 50 μL stop solution. The OD_405_ was measured on a microplate reader. An endotoxin concentration of less than 0.2 EU mL^−1^ was considered qualified for DCs infusion.

##### Flow Cytometric Analyses of Human DCs Products

The DCs for infusion were tested for the percentage of live cells (live/dead staining), purity (CD3^−^CD80^+^HLA‐DR^+^ cells in live cells), and maturation (CD83^+^ in DCs) by using flow cytometric analysis at day 7. Briefly, the cells were stained with live/dead fluorochrome for 30 min at 4 °C, washed with PBS, and incubated with a mixture of antibodies against surface markers (CD3, CD80, and HLA‐DR; the antibody details are provided below) for 30 min at 4 °C, washed by and resuspended in PBS containing 2% PBS, and then analyzed by flow cytometry LSRFortessa (BD Biosciences). A preparation with live/dead cell percentage higher than 90%, DCs purity more than 60%, and maturity rate higher than 50% was considered qualified for infusion.

For patient 6, the PBMCs were isolated from freshly heparinized blood of treated patients by Ficoll density centrifugation. The cells were stained with live/dead fluorochrome for 30 min at 4 °C, followed by washing by PBS, and incubated with a mixture of antibodies against surface markers (CD3, CD4, CD38, HLA‐DR, CD69, CD153, PD1, KLRG1; detailed information on antibodies is provided below) for 30 min at 4 °C, followed by washing, fixation, and permeabilization with the fixation/permeabilization solution kit (BD Biosciences, 554 715) for 20 min at 4 °C. The fixed cells were then mixed with antibodies against intracellular cytokines (IFN‐γ, IL‐2, IL‐17, and TNF‐α; the detailed information of antibodies was provided below) and incubated for another 30 min at 4 °C and then analyzed by flow cytometry LSRFortessa (BD Biosciences).

##### Ex Vivo Stimulation of Cytokine Release from Human PBMCs by Mtb Antigen and by DCs Harboring Mtb Antigens

PBMCs were isolated from freshly heparinized blood from active TB patients by Ficoll density centrifugation. The isolated cells were washed with PBS 3 times and counted. Half of the cells were cryopreserved in liquid N_2_, and the other half were subjected to DCs differentiation in the presence of rhGM‐CSF and rhIL‐4, as described above. An *Mtb* antigen, chimeric antigen AB9166, containing *Mtb* immune dominant antigens Ag85A, Ag85B, and four latency‐associated antigens, Rv2029c, Rv2031c, Rv2626c, and Rv3126c, were added at day 5, and then rhTNF‐α was added to promote DCs maturation at day 6. At day 7, the cryopreserved PBMCs were thawed and counted and then incubated with antigen‐loaded DCs at the ratio of 5:1 for 24 h in a U‐bottom 96‐well cell culture plate in duplicate. Wells for direct stimulation with *Mtb* antigen AB9166 and unstimulated wells were included as control groups, respectively. The culture supernatant was collected to detect IFN‐γ by human IFN‐γ ELISA kit according to the manufacturer's instruction (eBioscience, 88‐7316‐76).

##### Antibodies

The antibodies used in the murine experiments were: CD11c‐Percp Cyanine 5.5 (clone N418), CD80‐PE‐Cyanine 7 (clone 16‐10A1), CD83‐FITC (clone Michel17), CD86‐eFluor 450 (clone GL1), MHC I‐PE (H‐2Kd; clone SF1‐1.1.1), MHC II‐APC (I‐Ad; clone AMS‐32.1), CD103‐ PE‐eFluor 610 (clone 2E7), CD69‐Spper Bright 645 (clone H1.2F3), KLRG1‐Super Bright 702 (clone 2F1), CD3‐eFluor 450 (clone 17A2), CD4‐APC‐eFluor 780 (clone RM4‐5), CD44‐FITC (clone IM7), CD62L‐Percp Cyanine 5.5 (clone MEL‐14), IFN‐γ‐APC (clone XMG1.2), IL‐2‐PE (clone JES6‐5H4), and TNF‐α‐PE‐Cyanine 7 (clone MP6‐XT22) from eBioscience, and CXCR3(CD183)‐BV510 (clone CXCR3‐173) from BD Biosciences, and IL‐17 A‐Alexa Fluor 700 (clone TC11‐18H10.1) from BioLegend.

The antibodies used in the human experiments were: CD3‐Percp Cyanine 5.5 (clone OKT3), CD80‐FITC (clone 2D10.4), CD83‐PE (clone HB15e), HLA‐DR‐APC (clone LN3), HLA‐DR‐PE‐Cyanine 7 (clone LN3), CD4‐FITC (clone OKT4), TNF‐α‐PE‐eFluor610 (clone MAb11), CD38‐APC‐eFluor 780 (clone HIT2), CD69‐Super Bright 780 (clone FN50), KLRG1‐APC (clone 13F12F2), and the live/dead fluorochrome fixable viability dye eFluor 455UV from eBioscience, and PD1‐BV650 (clone NAT105), IFN‐γ‐Pacific Blue (clone 4S.B3), IL‐2‐BV510 (clone MQ1‐17H12), and IL‐17 A‐ Alexa Fluor 700 (clone BL168) from BioLegend, and CD153/CD30L‐PE (clone 116 614) from R&D systems.

##### Statistical Analysis

The statistical analysis was performed using GraphPad Prism 9.0 software. The Student's *t*‐test was used to compare the statistical difference between the two groups, the one‐way ANOVA was used to compare multiple groups, and the two‐way ANOVA was used for the grouped analysis. Differences with *p < 0.05, **p < 0.01, ***p < 0.001, or ****p < 0.0001 were considered statistically significant.

## Conflict of Interest

Z.‐D.H., X.‐Y.F., J.W., X.‐H.L., and S.‐H.L. are co‐inventors on a patent application.

## Author Contributions

**Zhidong Hu**: Conceptualization (lead); Data curation (equal); Formal analysis (lead); Funding acquisition (lead); Investigation (lead); Writing—original draft (lead). **Xuhui Liu**: Data curation (equal); Formal analysis (equal). **Jing Wang**: Data curation (equal); Formal analysis (equal). **Ling Gu**: Investigation (equal). **Zhenyan Chen**: Investigation (equal). **Lu Xia**: Resources (equal). **Heng Yang**: Investigation (equal). **Jinchuan Xu**: Investigation (equal). **Xuejiao Huang**: Investigation (supporting). **Huiling Wang**: Investigation (supporting). **Shuihua Lu**: Supervision (lead). **Xiao‐Yong Fan**: Conceptualization (lead); Funding acquisition (lead); Supervision (lead); Writing—review & editing (lead). **Zhidong Hu**, **Xuhui Liu**, and **Jing Wang** contributed equally to this work.

## Supporting information

Supplementary Material

## Data Availability

Research data are not shared.
