# Supplementary material for: Dendritic Cell Vaccine Harboring Inactivated Mycobacteria Induces Immune Protection Against Tuberculosis in Murine Models and is Well Tolerated in Humans
Source: Small Sci. 2024 Dec 18;5(2):2400355. doi: 10.1002/smsc.202400355 (PMC11934892; doi:10.1002/smsc.202400355)
Supplement: Supplementary file 1 — Supplementary Material [file SMSC-5-2400355-s001.pdf]

Supplementary Figures

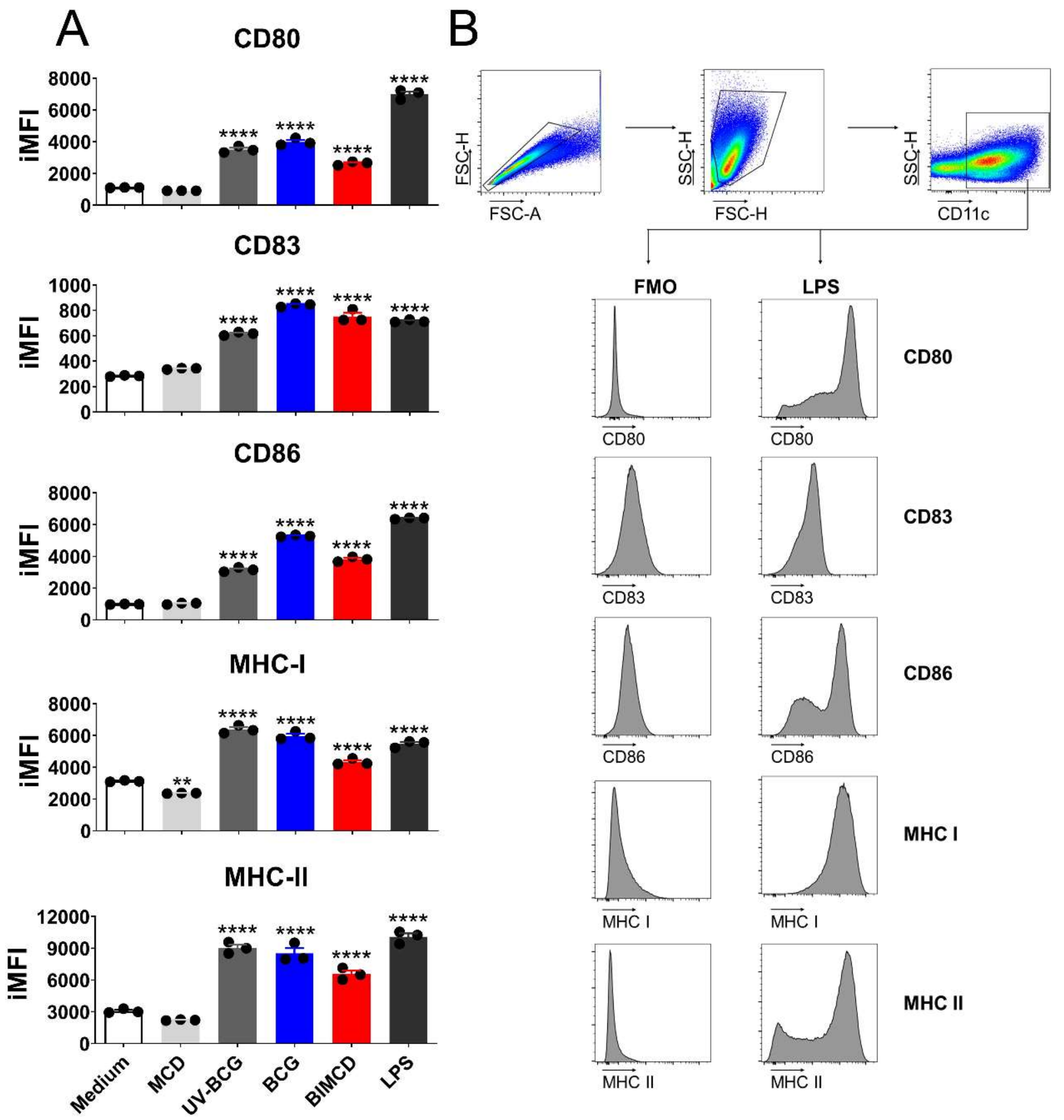

Figure S1. The gating strategy of flow cytometric analysis in DCs activation experiments and iMFI analysis. Representative flow cytometry histograms are shown. Briefly, DCs were defined as CD11c^{+} cells and the expression of surface markers on CD11c^{+} cells was detected with appropriate antibodies (n=5, one-way ANOVA). The iMFI values are shown in (**A**), and the gating strategy is shown in (**B**). These results are representative of three independent experiments with three replicate wells per group. Values are expressed as mean ± SEM. ****P <0.0001.

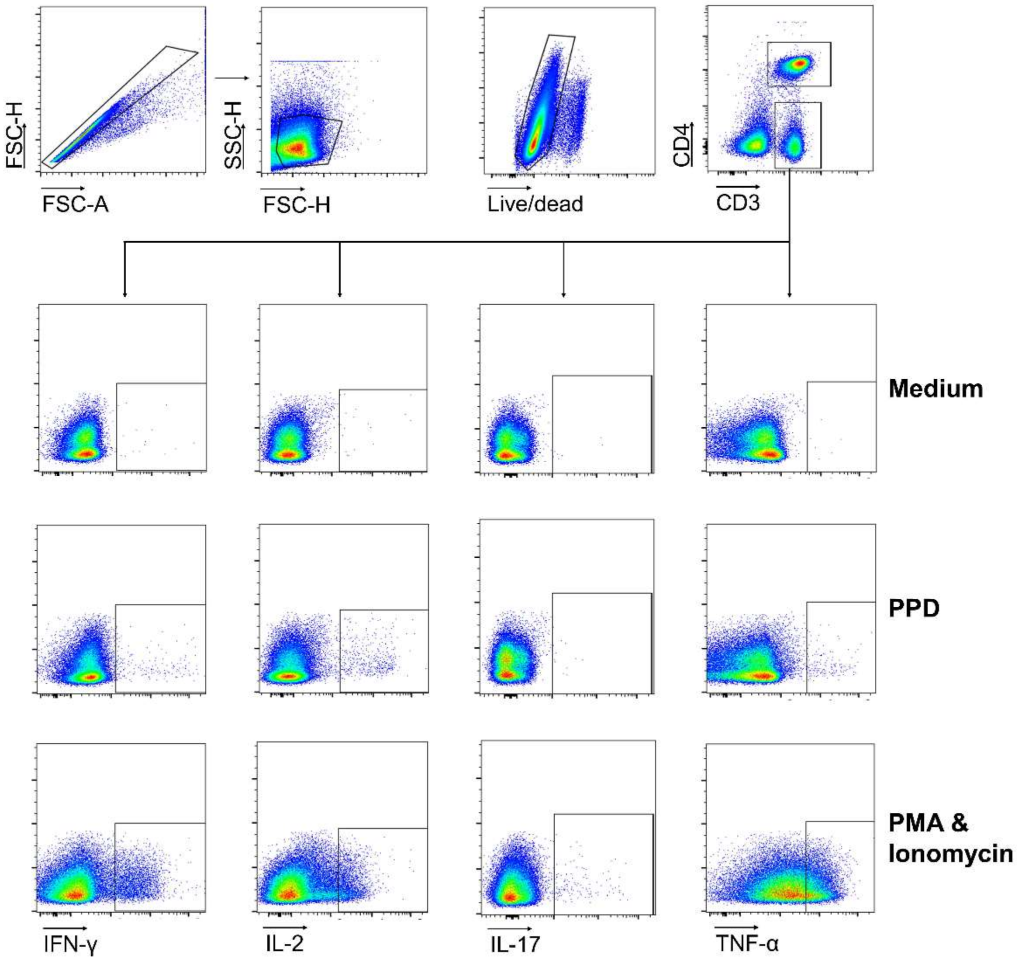

Figure S2. The gating strategy of intracellular staining in flow cytometric analysis in mouse experiments. Representative flow cytometric plots are shown. Briefly, The CD4 T cells were gated as $CD3^{+}CD4^{+,}$ and CD8 T cells were defined as $CD3^{+}CD4^{-}$. The cells were stimulated with PPD, with the medium as the negative control and PMA plus ionomycin stimulation as the positive control. The expression of intracellular cytokines was detected with appropriate antibodies.

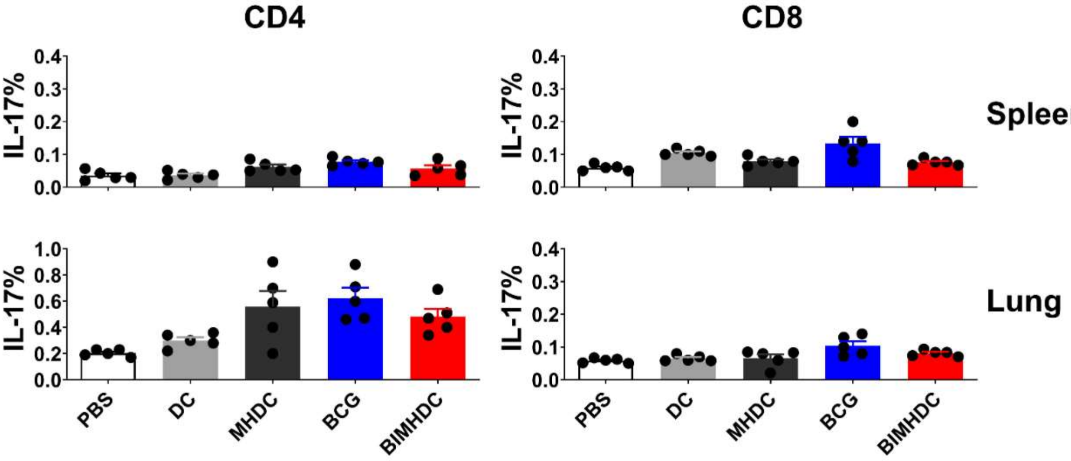

Figure S3. The antigen-specific Th17 immune responses in T cells. The splenocytes or lung cells were stimulated with PPD, with the medium as negative control. The expression of IL-17 in CD4 and CD8 T cells was detected by ICS assay (n=5, one-way ANOVA). The results are representative of two independent experiments with five mice per group. Values are expressed as mean ± SEM.

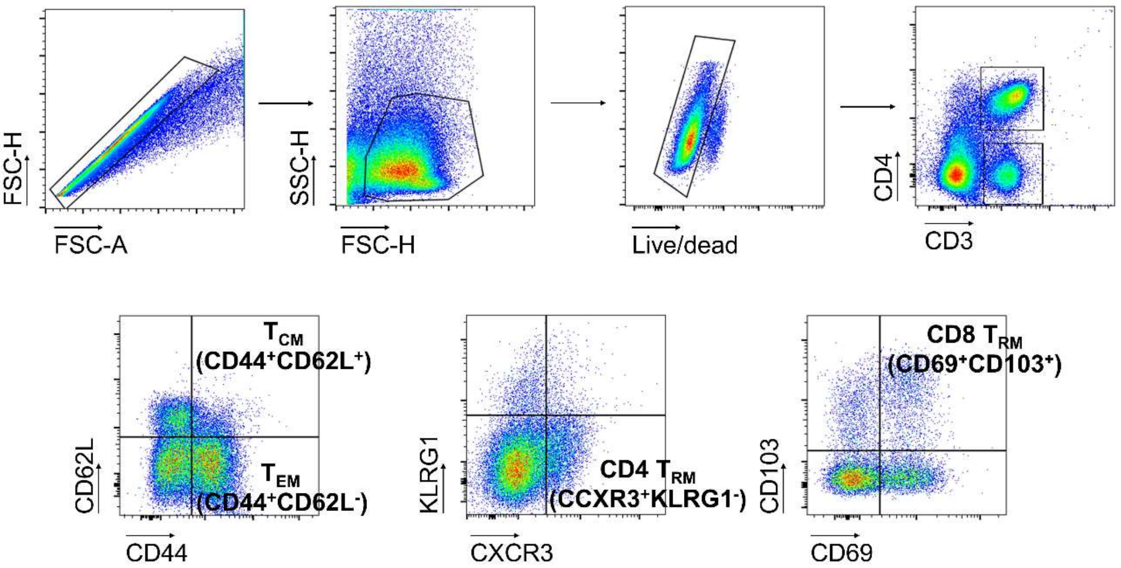

Figure S4. The gating strategy for memory phenotypes of T cells. Briefly, The T_{CM} and T_{EM} were defined as $CD44^+CD62^+$ and $CD44^+CD62^-$, respectively. The CD4 and CD8 T_{RM} were defined as $CXCR3^+KLRG1^-$ and $CD69^+CD103^+$, respectively.

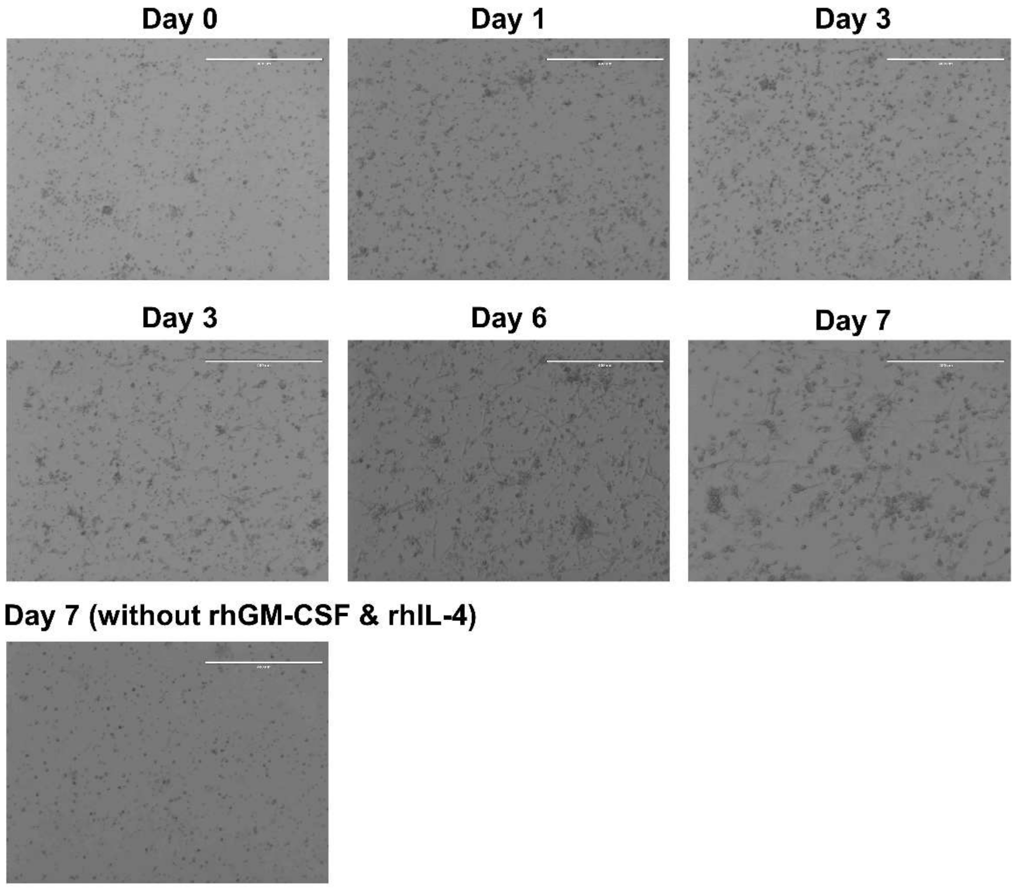

Figure S5. Light microscopy showing human DC differentiation.

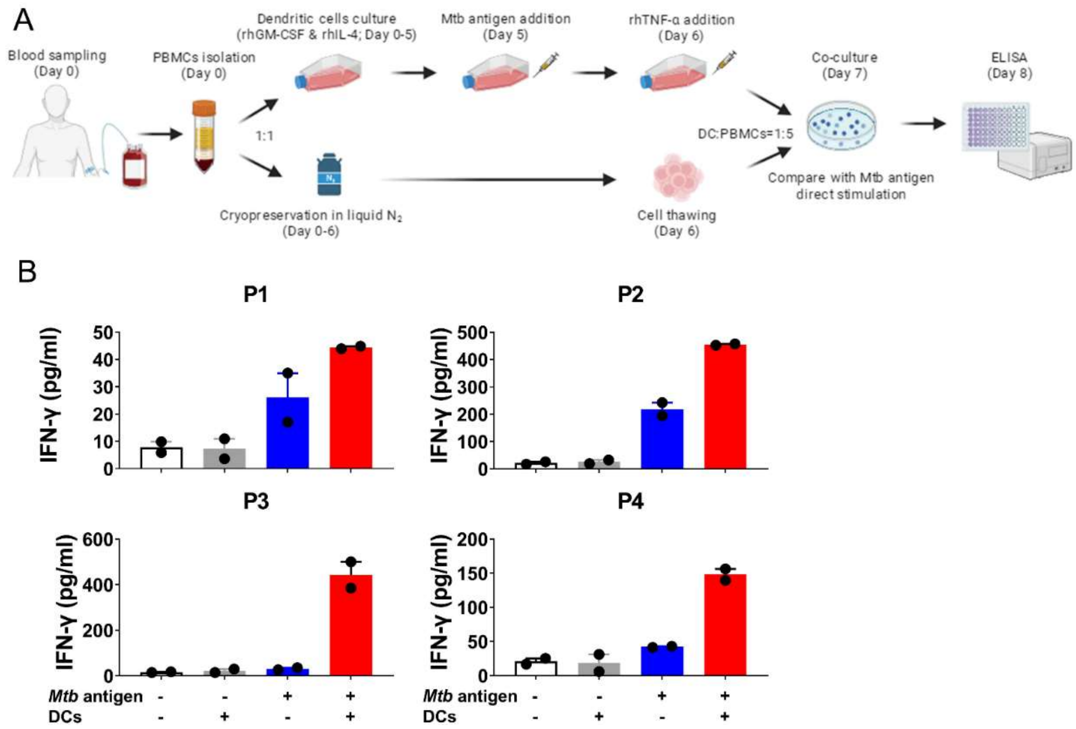

Figure S6. Preliminary *in vitro* stimulation experiments showing that DCs addition enhanced the antigen-specific T-cell immune response of TB patient's PBMCs. (**A**) Flow diagram of the experiments. (**B**) Antigen-specific IFN-γ responses induced by *Mtb* antigens, by DCs harboring *Mtb* antigens, or by the combination (n=2, one-way ANOVA). P1 to P4 represent four individual active TB patients, with duplicate wells per group.

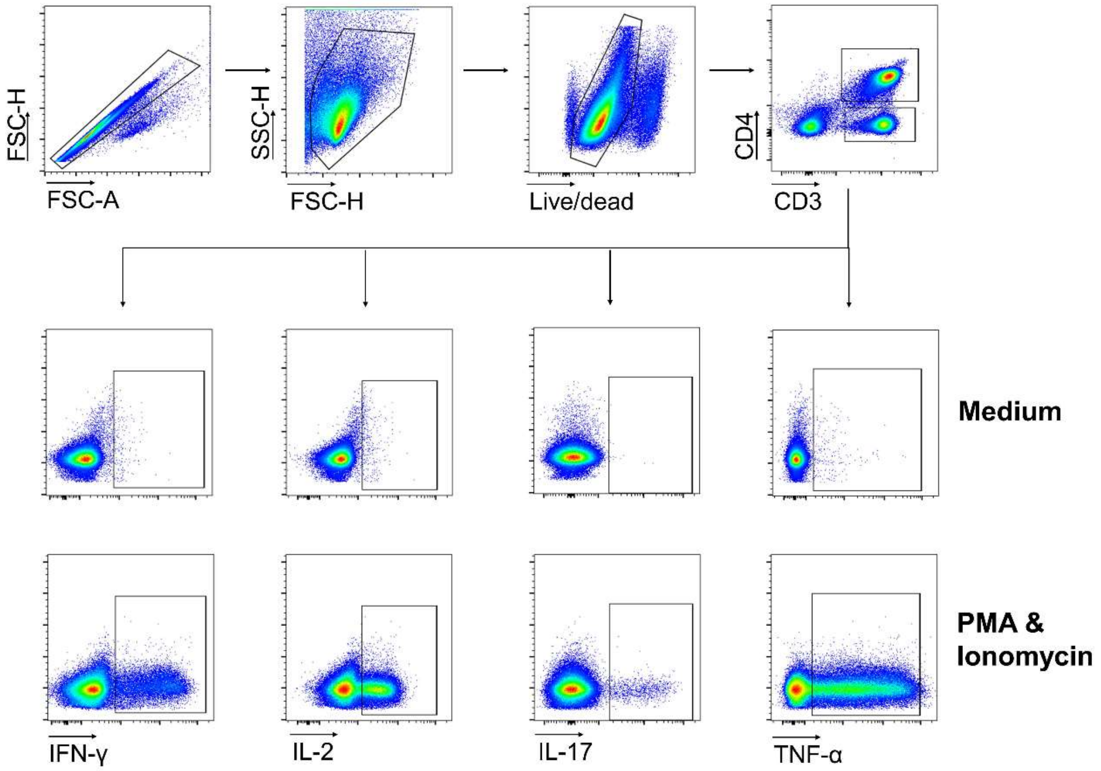

Figure S7. The gating strategy of intracellular staining in flow cytometric analysis in human PBMCs. Representative flow cytometry plots are shown. Briefly, The CD4 T cells were gated as $CD3^{+}CD4^{+}$, and CD8 T cells were defined as $CD3^{+}CD4^{-}$. The expression of intracellular cytokines was detected with appropriate antibodies.

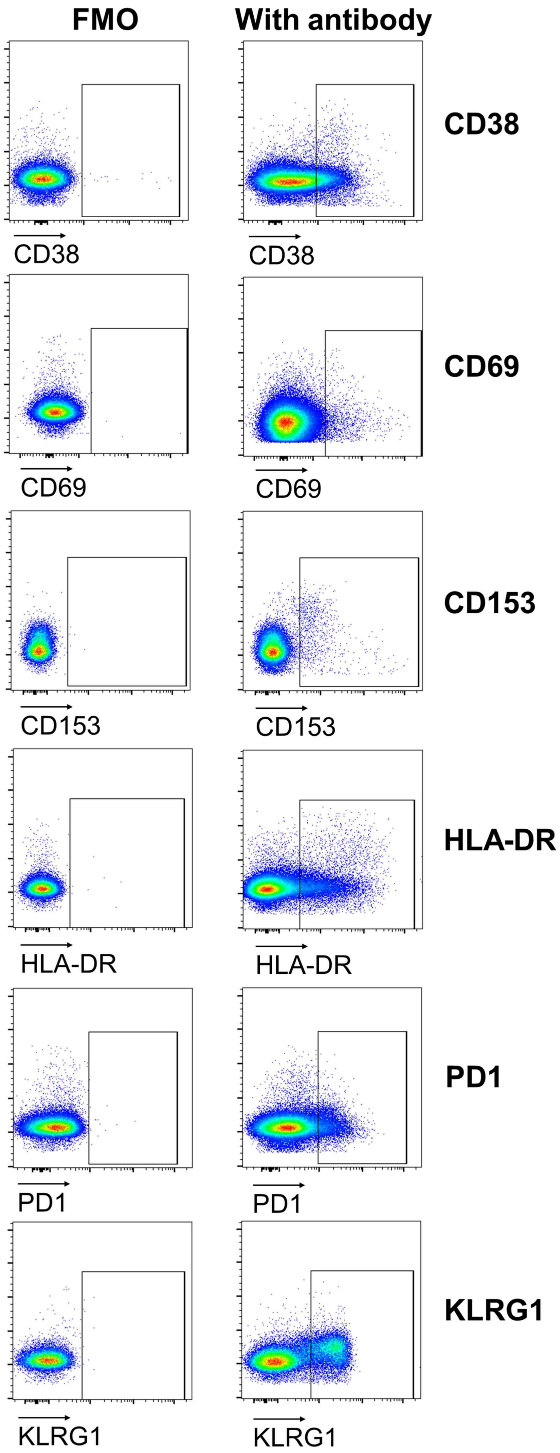

Figure S8. The gating strategy of surface makers staining in flow cytometric analysis of human PBMCs. The expression of surface markers expression on CD4 and CD8 T cells was detected with appropriate antibodies. The fluorescence minus one (FMO) control was used to guide the gating of corresponding surface markers.
